# Disposable Paper-Based Biosensors for the Point-of-Care Detection of Hazardous Contaminations—A Review

**DOI:** 10.3390/bios11090316

**Published:** 2021-09-04

**Authors:** Mohammad Mahdi Bordbar, Azarmidokht Sheini, Pegah Hashemi, Ali Hajian, Hasan Bagheri

**Affiliations:** 1 Chemical Injuries Research Center, Systems Biology and Poisonings Institute, Baqiyatallah University of Medical Sciences, Tehran 19945, Iran; mohammadmahdibordbar@gmail.com; 2 Department of Mechanical Engineering, Shohadaye Hoveizeh Campus of Technology, Shahid Chamran University of Ahvaz, Dashte Azadegan 78986, Iran; azar.sheini@gmail.com; 3 Research and Development Department, Farin Behbood Tashkhis Ltd., Tehran 16471, Iran; pegi.hashemi@gmail.com; 4 Institute of Sensor and Actuator Systems, TU Wien, Gusshausstrasse 27-29, 1040 Vienna, Austria; ali.hajian@tuwien.ac.at

**Keywords:** paper sensors, toxic substances, biological receptors, optical detection, electrochemical methods, rapid tests

## Abstract

The fast detection of trace amounts of hazardous contaminations can prevent serious damage to the environment. Paper-based sensors offer a new perspective on the world of analytical methods, overcoming previous limitations by fabricating a simple device with valuable benefits such as flexibility, biocompatibility, disposability, biodegradability, easy operation, large surface-to-volume ratio, and cost-effectiveness. Depending on the performance type, the device can be used to analyze the analyte in the liquid or vapor phase. For liquid samples, various structures (including a dipstick, as well as microfluidic and lateral flow) have been constructed. Paper-based 3D sensors are prepared by gluing and folding different layers of a piece of paper, being more user-friendly, due to the combination of several preparation methods, the integration of different sensor elements, and the connection between two methods of detection in a small set. Paper sensors can be used in chromatographic, electrochemical, and colorimetric processes, depending on the type of transducer. Additionally, in recent years, the applicability of these sensors has been investigated in various applications, such as food and water quality, environmental monitoring, disease diagnosis, and medical sciences. Here, we review the development (from 2010 to 2021) of paper methods in the field of the detection and determination of toxic substances.

## 1. Introduction

One of the major challenges in developed countries is the uncontrolled spread of hazardous contaminations, due to the activity of industrial centers or microorganisms. They can be classified by source (plant, animal, mineral, or chemical agents), nature (metal, toxin, microorganism, or organic compound), and their uses (insecticides, food additives, or fungicides). The contaminations are classified in the chemical, environmental, agricultural, medical, and radioactive categories [1]. Among them, the environmental and agricultural contaminations were considered in this review. These contaminants are different in nature: chemical (metal and organic compounds), biological (pathogen bacteria and virus), and physical (energy) [1]. Focusing on the chemical and biological compounds, these contaminants can affect the ecosystem of an area by penetrating water, soil, and air remaining in the environment and entering the human life cycle through inhalation, skin absorption, and swallowing [2]. Depending on the toxicity degree and contamination exposure duration, the toxic substances can result in different influences on human health. These influences may be temporary, leading to headaches, nausea, lung failure (due to the inhalation of volatile gases), blood poisoning, liver and kidney failure, and even cardiovascular failure [3]. Moreover, high doses of toxins may weaken the immune system, as observed for compounds such as carbon disulfide, mercury, manganese, arsenic, lead, and cadmium [4,5,6]. On the other hand, some compounds (e.g., aflatoxins and organophosphates or some metals, such as lead and cadmium) may remain in the body for a long time and their excretion process may be prolonged, leading to nervous system dysfunction [7].

Since toxins with extremely low concentrations can also pose serious hazards, it is important to detect them using a sensitive method. In fact, the detection is carried out in a real sample consisting of thousands of chemical species. Therefore, the detection method needs to be highly selective, identifying the species in the presence of similar compounds. The detection analysis is performed with large and small analytical devices. The former is based on chromatographic methods (e.g., high-performance liquid chromatography and gas chromatography) or spectroscopic methods (e.g., infrared, ultraviolet (UV)-visible, mass, and nuclear magnetic resonance). These methods provide a unique response for each real sample, thereby detecting the presence of the desired analyte in the sample and quantifying its concentration. Moreover, the detection methods are able to measure extremely small amounts of toxic species. Nevertheless, their measurement process is time-consuming and expensive. Meanwhile, analyzing and interpreting the results requires a skilled operator and sufficient knowledge [8].

Alternatively, the small devices makes it possible to perform analytical experiments using the lowest volume (up to picomolar levels) of indicators and analytes [9]. In turn, this makes the analysis process rapid and cost-effective, as the small devices are not complex and do not require special laboratory conditions [10,11]. Therefore, it is possible to use them in the sampling sites and by individuals who need the analysis. Mostly, the detection methods based on these devices are called point-of-care test (POCT) [12], being widely used in diagnosing diseases, examining food control, monitoring environmental pollution, etc. [13]. The commercial types of POCTs are available in the market such as diagnosing prostate cancer, intestinal cancer, infectious diseases, pregnancy diagnosis, drinking water quality control, detection of food spoilage, food adulterations, etc. [14,15]. Profits from the production of POCTs are expected to reach 39.96 billion dollars by the end of 2021 [16]. Basically, POCTs consist of different parts: the sensor substrate, the sample inlet, the receptor, and the response transducer. Receptors can be chemical compounds (e.g., inorganic complexes, organic markers, polymers, and nanoparticles) or biological species (e.g., antibodies, antigens, aptamers, enzymes, or part of a plant or animal tissue).

In terms of selectivity, bio-POCTs outperform chemical POCTs [17]. Bioreceptors respond mainly to a specific analyte, thus increasing the sensor’s ability to determine a species in the presence of additives and other contaminants [17]. The responses generated by bio-POCTs are accurate and reliable, determining extremely low concentrations of the analyte [18]. Nevertheless, compared to chemical POCTs, they need special storage conditions, in a narrow range of parameters, or they suffer from the complexity of the storage process [19]. The activity of the bioreceptors are reduced by mechanical and environmental changes, thus leading to the inefficiency of the resultant sensor [19]. Furthermore, the cost of fabricating biosensors is much higher than that of a chemical sensor [19].

**Figure 1:**
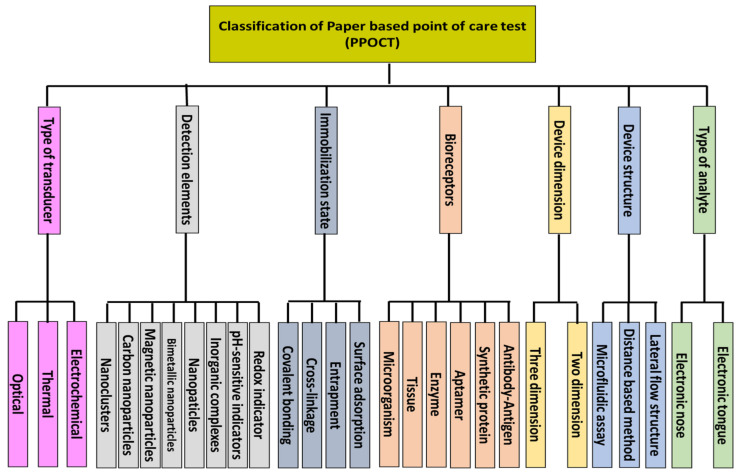
Schematic diagram for PPOCT classification, which is described in this review.

However, in recent years, the development of bio-POCTs has increased in both the laboratory and commercial fields [16]. In this study, we will review different types of bio-POCTs, while also investigating their application in detecting and determining hazardous contaminations, such as mycotoxin, organophosphate, bacteria, and heavy metal ions. This category has not been reported in previous studies. Figure 1 shows an overview of the bio-POCTs classification in this study.

## 2. Bio-POCT

To design a bio-POCT, a sensing element (being primarily a biological compound) is initially coupled to a detection element. A transducer is connected to the detection element, converting its changes into an intelligible signal. This structure can be installed on a well-plate or substrate. It is clear that performing a well-plate test requires a time-consuming preparation process, as well as a skilled operator for the laboratory conditions and tools, for a typical test. Additionally, moving the designed device for on-site analysis is cumbersome. The components of a bio-POCT can be immobilized on a substrate, providing a portable structure, which requires low amounts of materials for performing a test.

### 2.1. Substrates Used in the Bio-POCT

To select the substrate, different features, including flexibility, biocompatibility, biodegradability, availability, cost-effectiveness, surface modification, permeability, and portability of the sample are considered [20]. To this end, substrates such as glass, polydimethylsiloxane (PDMS), silicone, and paper have been used [21]. Among them, the use of paper as a substrate is very popular, due to its fibrous structure, enabling us to easily modify it [22]. Moreover, paper has a capillary nature, making the liquid sample flow easily on the substrate, while also providing the possibility of the penetration of gaseous samples into its textures [23]. The paper selected as the substrate must be so flexible that it does not break or tear when fabricating sensors with a three-dimensional (3D) design [24]. This substrate should have a thickness of 10–100 µm, consuming less volume of the sample (in the microliter level) [25].

The paper substrate needs to have a soft texture that can be easily attached to solids, collecting small amounts of the sample [26]. Additionally, it needs to be a strong absorber, storing an exact volume of a sample for the subsequent displacement of a chemical [27]. The paper substrate should also be permeable to air and gas, with a network structure, in order to separate the analyte from the contaminated matrix, by filtering disturbing components [28]. By having a high surface-to-volume ratio, the paper substrate is capable of immobilizing a large number of sensing elements on its surface [29]. Moreover, it should be compatible with biological samples. In some cases, applying heat treatments may be required to immobilize the enzyme or antibody coated on the nanoparticles [30]. Thus, the paper substrate must be heat-resistant [31]. Overall, it should be inert against physical and chemical changes [32].

### 2.2. Types of Paper

To select paper as a substrate in the preparation of a paper-based POCT (PPOCT), the following three factors must be considered: the purpose of the determination, the specificity of the analyte, and the characteristics of the measurement method. Paper substrates should be selected in such a way as to play a positive role in the sensitivity, selectivity, and reduction of the interfering effect on the method response and the stability of the sensor. Depending on the purpose of the study, the paper substrate can vary, in terms of thickness, pore size, permeability, and capillary nature, as well as the flow rate of the sample on the substrate, smoothness, and softness [33].

In recent years, most paper-based sensors have been made of cellulose substrates [34]. The most popular cellulosic substrates are filter, chromatography, and blotting papers, which have different grades [35]. For example, Whatman grade 1 filter paper is made of 98% cellulose, with a uniform and smooth surface and a thickness of 0.18 mm. Liquids flow on these papers at moderate speeds. They also have pores with a size of 0.11 μm. As an adsorbent substrate, the retention rate for this paper is fine. Higher grades of Whatman paper have larger pore sizes, causing the increase in the sample retention rate. For example, Watman grade 4 paper has a pore size of 25 μm. The weight of Whatman filter paper changes from 85 to 100 g·m^−2^, based on the grade of the paper [36].

Cellulose papers have a specific surface area of 1.4 m^2^.·g^−1^. The porosity of the papers is high. It has good hydrophilic properties, mechanical strength, and is easily degradable [37]. One way to create cellulose substrates is to use bacteria. Compared to other cellulose substrates, bacterial cellulose has specific advantages, such as renewability and biocompatibility. The paper porosity increases up to 92% [37].

The properties of cellulose paper can be changed by adding some compounds, such as surfactants, polymers, aldehydes, and epoxy groups [38,39]. Notably, the hydrophilicity, porosity, retention of the sample, and the flow rate of liquid on the paper varies, depending on the type and amount of modifiers [40]. Cellulose surface modification can improve the physical and chemical properties of the paper substrate. For example, surface area and adsorption capacity can be increased to 172.49 m^2^.·g^−1^ and 158.98 mg.·g^−1^, respectively [37].

It is possible to produce nitrocellulose by nitrating cellulose partially [37]. The nitration process increases the porosity and hydrophobicity of cellulose, forming membranes for suitably immobilizing biological species, such as enzymes, proteins, and antibodies [37]. Nevertheless, they have a more fragile structure than cellulose substrates [37]. Here, an electrostatic interaction occurs, in which positively charged biological species are adsorbed to the negatively charged surface of nitrocellulose. The pore size of these papers varies from 9 to 55 μm. Additionally, the porosity rate is in the range of 75 to 81%, depending on the type of paper. Using nitrocellulose papers, the absorption ability improves by up to 2038 mg.·g^−1^. Of course, this claim applies to substrates most commonly used in biosensor structures [37].

The glossy paper is another substrate used to prepare paper sensors. This type of paper is made of cellulosic fibers bonded to an inorganic material, giving rise to flexible, non-degradable, and relatively smooth substrates, whose surface can be easily modified with other compounds, such as nanomaterials [41]. In turn, the hydrophobicity of the resulting paper increases, which can be used mainly for colorimetric experiments [40]. Recently, nanocellulose has been used to produce transparent papers with very high aspect ratios, including cellulose nanofibers, crystalline nanocellulose, and bacterial nanocellulose [42]. The density and fiber resistance of the transparent papers increase, making them more resistant to moisture and heat [43]. In paper devices, the substrates should be as insensitive to ambient humidity as possible. Since the humidity changes during the day (or on different days), the use of hydrophobic polymer substrates is preferred, being inert in the relative humidity range of 10–100% [44]. Notably, polyethylene terephthalate, polyvinylidene difluoride, and polypropylene substrates show high chemical resistance to gases, acids, and bases [44]. These substrates have been used extensively to detect volatile species in the vapor phase [45].

## 3. Classification of Bio-PPOCT, Based on the Analyte Type

The analysis of samples can be performed in gas and liquid phases. In the gas phase, the goal is to detect volatile analytes existing naturally in the environment (e.g., air pollutants) or caused by the decomposition of a material. Volatile compounds are mainly analyzed as metabolites in exhaled breath [46], sweat [47], and saliva [48] secretions, as well as blood [49] and urine vapors [50], diagnosing a disorder in the body. These compounds can also be formed from the breakdown of proteins, fats, and carbohydrates in food, so that they can be used to control the quality of food products [51,52,53,54]. It is even possible to determine the amount of impurities present in petroleum products, supplements, and medicines qualitatively and quantitatively [44]. Of late, bacteria and fungi have been identified based on their volatile compounds [55,56]. In this regard, the paper sensor is exposed to the sample vapors, having a high chemical and mechanical resistance to moisture, along with good permeability to penetrate the analyte in its paper texture, in order to interact with the indicators [44]. The pore size, thickness, and surface to volume ratio of the paper are the considered factors to have a practical substrate for storing the vapors [57]. The resulting devices can contain one or more sensors, being capable of detecting one or more gas samples. Since their performance is similar to the olfactory system, the sensor devices are called electronic noses [58].

In the liquid phase, the analyte is either a pure liquid or a component dissolved in a solution. In this respect, the purpose of analysis is the diagnosis of a disease, detection of an environmental pollutant, evaluation of a food product, and so on [34]. To analyze the samples in the liquid phase, the paper is either immersed in the liquid sample or part of the sample is injected into the surface of the paper [59]. In the latter case, the sample is transferred to the detection zones through embedded channels or moves along the paper strip (arising from the capillary nature of the paper) to react with the indicator [59]. Accordingly, the paper with high hydrophilicity and low permeability should be chosen [23]. Moreover, the flow rate of the sample should be appropriate for transporting the liquid samples from injection zones to detection ones [23], also allowing for the interaction between the analyte and the indicator [23]. These sensors mostly use Whatman grade 1 paper [60]. In this case, the analysis can be single species or multispecies, having a structure similar to the taste system, which is the so-called electronic tongue [61]. One important point that must be considered in the fabrication of these sensors is the lack of displacement of the sensing element. To this end, the surface of the detection zones is modified with polymeric compounds (e.g., chitosan and polyvinyl alcohol) or protein compounds (e.g., BSA), maintaining the indicator stationary on the surface. It is also possible to mix the indicator with hydrophobic or plasticizing compounds, in order to fix it on the paper surface without having a negative effect on the sensing ability and sensitivity of sensor [62,63].

## 4. Classification of Bio-PPOCT, Based on the Device Structure

The simplest configuration for a paper device is the dipstick, in which the sensor is immersed in the solution, in order to detect analytes [64]. This device is mostly used for qualitative detection and employed as paper strips sensitive to medium pH, urinary infections, metabolites, urinary proteins, and water contaminants [65]. While the design of these sensors is apparently simple, the reagents must be placed on paper and do not leak into the solution during immersion. Moreover, the species suspended in the solution should not be adsorbed on the texture of the sensor, thus obtaining the sensor response efficiently. However, the use of dipstick devices is limited, partly due to the high adhesion and viscosity of the liquid. Sometimes the goal of the study is the determination of several analytes simultaneously; therefore, several detection reagents should immobilize on the surface of paper without merging together. In some other cases, appropriate reagents may not be available (thus making it necessary to convert the analyte to another species) or it is not possible to carry out the analysis, due to the presence of foreign species. Therefore, a series of preparatory processes are required to be performed on the sample prior to the measurement. The dipstick design does not address these limitations.

### 4.1. Lateral Flow Structure

**Figure 2:**
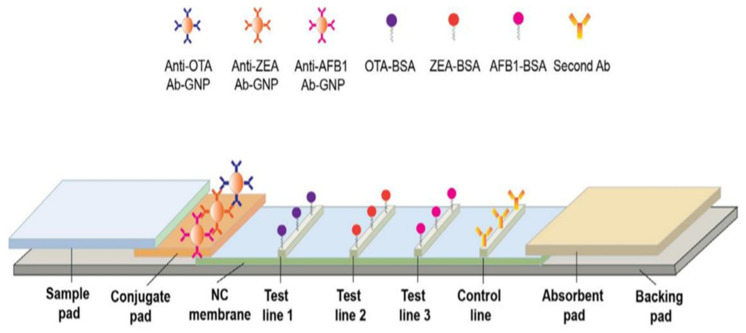
Different components of a lateral flow structure. This structure consists of sample pad for receiving the sample, conjugated pad for interacting analyte, and labeled detection element; detection pad containing test lines for forming complexes between capture bioreceptor and labeled analyte and absorbent pad for terminating the sample flow. The gray rectangular shows the back pad. This schematic was proposed by Chen et al., for the simultaneous determination of aflatoxin B1, zearalenone, and ochratoxin A in agriculture products (reprinted with permission from [68]; copyright (2016) Elsevier).

The lateral flow structures complies with the principles of enzyme-linked immunosorbent assays (ELISA), made of rectangular paper strips that are typically a width of 6 mm and length of 7 cm [66]. The sample moves along the paper on a series of consecutive pads [66]. As illustrated in Figure 2, the four main components of these sensors are the sample pad, conjugate pad, detection pad, and absorbent pad (embedded along the paper strip) [66]. The sample is injected into the sensor through the sample pad, storing a large part of the liquid, while also directing it to the conjugate pad [67].

In order to improve the performance of the sensor, the sample pad is pre-treated using a buffer solution, at a certain pH and ionic strength, before adding the sample. The interaction between the analyte and the receptor can be influenced by the pH and ionic strength [69,70,71]. To prevent non-specific interactions between the sample and the paper, while also facilitating the sample transfer, the sample pad is modified by detergents, such as SDS, Tween 20, and Triton [67]. Blocking agents such as BSA or Casein can even be used to eliminate non-specific bonds [67]. In order to remove microbial contamination, the sample pad is impregnated with sodium azide [67]. Sometimes, a filter is placed on the sample pad to remove the interfering species containing analytes, including proteins and blood cells [72]. The thickness of the paper should be taken into consideration when choosing a sample pad. In other words, the thicker the paper, the slower and more stable the flow [72]. Since the pad may affect the measurement target, it needs to be free of chemical impurities. The sample pad can be made of cellulose fibers or glass fibers [67]. While the cellulose fibers are thicker and cheaper than the glass ones, they are difficult to handle. In contrast, glass fibers with good tensile strength are capable of uniformly distributing the sample on their surface, thereby acting as a filter. Nevertheless, glass fibers with lower cutting ability are more expensive than paper fibers and can be contaminated with environmental chemicals during the fabrication process [73].

On the other hand, the conjugate pad is made of nitrocellulose, immobilizing the bioreceptor on its surface [74]. This pad can formed by the other membranes such as nylon, polyvinylidene fluoride, or polyvinylidene difluoride membranes, but nitrocellulose is more attractive because of some of its advantages, such as having a high capacity for immobilizing the biological compounds and having low costs [75]. In this case, the target analyte (e.g., antigen) interacts with the bioreceptor (e.g., antibody). The solution is directed to the conjugated pad, based on the capillary nature of the paper. The bioreceptor is conjugated to color compounds with unique optical and electrical properties [74]. These compounds mainly comprise of carbon dots, as well as metal (e.g., Au), upconversion, and magnetic nanoparticles [67]. Among them, Au nanoparticles (AuNPs) produce a stable red color (being observable with the naked eye), show good physicochemical stability that can be easily functionalized, and have low toxicity [76]. Thus, AuNPs are employed as a label on the conjugate pad. Since the nanoparticles used are colloidal suspensions and their stability is affected by the ionic strength of the solution, the conjugated pad is modified by a buffering agent [67]. For this purpose, some sugars, such as sucrose and trehalose, are mostly used [67].

In the conjugate pad, the target analyte interacting with the labeled bioreceptor creates a complex, moving toward the detection pad. Two lines are created on the detection pad: one is the test line and the other is the control line [77]. In the former, the capture bioreceptor interacts with the labeled analyte, indicating the existence of the analyte in the environment. In the latter, the correct performance of the designed system is evidenced [77]. The analyte detection is carried out on the detection pad, involving the two following principles: competitive or inhibitory methods and sandwich methods [67]. In the competitive method, the target in the sample competes with the one labeled in the conjugated pad to interact with the capture bioreceptor, mostly used for analytes with a small size and high concentration [78]. In contrast, the sandwich method is very popular for detecting medium- and large-sized analytes, including proteins, antibodies, cells, and bacteria [79]. In the sandwich method, the analyte is sandwiched between the detection (primary) and capture (secondary) bioreceptors in the test line. These bioreceptors can be both monoclonal. In some cases, the detection bioreceptor is monoclonal and the capture bioreceptor is polyclonal [67]. A high concentration of the capture bioreceptor in the test line is recommended for the sandwich method [67]. Finally, the absorbent pad is the last part of a lateral flow system with a sufficient bed volume, thereby terminating the sample flow [80]. The lateral flow components are pasted to a polymer substrate, via the help of a pressure-sensitive adhesive. This substrate, known as the backing pad, is mainly made of polystyrene or plastic materials. The strength and flexibility of the created strips depends on the material of this pad [75].

The flow velocity and pore size of the membrane affects the assay sensitivity in the lateral flow system. The high sensitivity is achieved via the strong interaction between the labeled analyte and test line antibody. For this purpose, the membranes should have a small pore size with a slow sample flow rate [81]. This prolongs the experiment time, which is between 10 and 30 min for a simple test. In addition, the lateral flow system of sandwich format suffered from the hook effect. The hook effect is a phenomenon in which free analytes in the media compete with the labeled analyte for binding to the test line antibody. This has a negative effect on the color intensities and, consequently, the sensor responses [81]. Flow-through immunoassay (immunofiltration assay) can be used to reduce these limitations. In the alternative assay, a larger sample volume is consumed, thus improving the kinetics and sensitivity. Hooke phenomenon is not observed in these methods. An immunofiltration assay can be performed by passive and alternative approaches. In the former, the lateral flow pads are layered by stacking method so that the detection pad is located on the top of the conjugate and absorbent pad. In the later, the membrane is embedded into the syringe filter holder after modification with a bioreceptor. Reagents and samples flow vertically over the membrane through the syringe [81].

### 4.2. Distance-Based Method

The method of stain length measurement uses another 2D strip structure, in which a strip of Whatman grade 1 paper (with dimensions of 0.5 cm × 3–5 cm) is used and impregnated with detection reagents [82]. The sample is added to the sensor from the injection site, moving along the sensor to react with the reagent, while also changing its color [49]. The distance moved is measured by a ruler and depends on the concentration of the analyte [83]. In this way, unlike the lateral flow methods, the entire analysis process is performed on a single pad with a simpler design, detecting small molecules. In addition to qualitative diagnosis, the stain length measurement can also be used for quantitative analysis [83].

### 4.3. Microfluidic Assay

Another widely used 2D configuration is microfluidic structures, in which an extremely small volume of the sample (in the range of 10^−6^–10^−18^ L) is consumed [84]. In these structures, the sample flows in channels with a width of 10 µm [84]. Certainly, the behavior of liquids at the micro-scale is different from that at the macro-scale and can be influenced by several factors, such as the surface tension and fluid resistance [84]. In this respect, the effect of surface forces is found to be greater than that of volumetric forces [85]. In microfluidic structures, all the components needed for the liquid entrance, pumps, valves, and mixers, along with the detection devices (i.e., transducers and indicators), are mounted on a very small substrate. Depending on the driving force of the liquid transfer, the substrate used, and the system configuration, microfluidic devices can be categorized as open microfluidic [86], continuous flow microfluidic [87], drop microfluidic [88], digital microfluidic [89], paper microfluidic [90], and microfluidic particle detection systems [91]. Among these devices, the microfluidic paper system, designed by Whitesides, enables the flow of the liquid on the surface of a porous substrate, based on the capillary nature [90]. With this system, no external driving force is required to transfer the liquid. In these sensors, the channels, the injection, and the detection zones can be created between hydrophobic barriers [90]. This matter can be one of the limitations of the paper-based methods because the width of the channels may be blocked by the hydrophobic barriers, so that only 50% of the actual sample volume may reach the detection zone. Despite all the limitations, paper microfluidic sensors are one of the most popular methods for fabricating the point-of-care instruments.

## 5. Classification of Bio-PPOCT Based on the Device Dimension

In fact, the configuration design is based on the direction of the sample flow on the paper [23]. This flow can be along the direction of the paper (i.e., the horizontal direction, forming a 2D configuration) [92] or along the depth of the paper (i.e., the vertical direction, creating 3D structures) [93,94]. To design each sensor structure, the desired pattern is drawn using design software, such as Photoshop, Illustrator, CorelDraw, InDesign, AutoCAD, etc. Accordingly, it is possible to shape and resize channels and detection zones with the above software.

### 5.1. Two-Dimensional Configuration

To have a 2D configuration sensor, the designed patterns should be executed on paper using physical or chemical methods [95].

#### 5.1.1. Physical Methods

Paper sensors are physically fabricated by the following four methods: knife plotter, craft cutting, embossing, and laser cutting [96].

In the knife plotter method, the cutting process was carried out by an automatic cutter and controlled by a computer program [97]. To prevent the paper from tearing, the cutting process was continuous, thus being performed in several consecutive steps [97]. The above-mentioned method is the simplest and least expensive one for preparing paper sensors. Additionally, the fabrication process was not time consuming, which enabled us to adjust the computer program to cut paper with different sizes and thicknesses [98]. This method was used only for the fabrication of hydrophilic areas. So, the complementary treatment using hydrophobic materials was necessary. Additionally, a large portion of the paper used was wasted [98].

In the craft cutting method, the paper is initially glued to a thin sheet, and the paper strips are then cut into different dimensions and sizes using a craft cutter [99]. The advantage of this method is the production of flexible, portable, and disposable substrates [100,101]. Sometimes the strips formed are modified by fluoroalkyl trichlorosilan, thereby increasing the hydrophobicity of the paper [96]. In turn, this reduces the capillary nature of the paper, while also requiring an external pump to transfer the liquid [100]. Additionally, it is not possible to create variable channels, based on the craft cutting method [96].

In the embossing method, the designed pattern is engraved on paper [102]. The paper is also moistened with ethanol and placed between two molds made of plastic. The surface of the paper can be modified by silane and sealed between two adhesive tapes, forming open channels with porous walls. This process creates flexible and foldable substrates, although they are prone to the absorption of contaminants, such as ambient gases, due to their porous structure [102].

Laser cutting is the last physical method in the fabrication of paper sensors, employing CO_2_ laser cutting to create the selected designs on paper [103]. This method is only able to cut a part of the paper thickness. The advantage of the laser cutting is the reproducible production of paper substrates in the shortest possible time, by using inexpensive tools. Similar to other physical methods, it is also prone to the absorption of environmental pollutants, due to the porous structure of the paper [104].

#### 5.1.2. Chemical Methods

In chemical methods, hydrophobic barriers are created by blocking holes in the paper. The most widely used chemical methods can be categorized as follows: photolithography, wax printing, inkjet printing, laser printing, flexographic printing, stamping, chemical vapor deposition, screen printing, and spraying [105].

The general chemical method is photolithography, in which the paper is initially impregnated with a photoresist (e.g., SU-8) and then exposed to UV light to selectively engrave the pattern on it [106]. The photoresist used in the engraved pattern is removed using organic solvents, such as propylene glycol monomethyl ether acetate and propanol [96]. The rest of the photoresist is removed with the help of oxygen plasma [90]. In this way, channels (with a width in the range of 80–200 µm) and hydrophilic zones are created between hydrophobic barriers, facilitating the flow of the liquid in them. One can also use TiO_2_ nanoparticles and light-sensitive polymers, mixed with silane instead of SU-8 [107,108]. Despite all of its advantages, the photolithography method requires expensive equipment and reagents, while also having a complex testing process. Additionally, the photoresists used have low mechanical resistance and can be cracked and broken [109]. In order to overcome these limitations, a simple UV lamp and a heating plate can be used, along with flexible UV-sensitive materials [33].

Another alternative approach is to use wax instead of photoresist compounds. Wax can be immobilized on paper using a pen, printer, or a metal mold [110,111]. Note that the placement of the wax is based on the designed pattern. By heating the paper, the wax penetrates the texture of the paper, closing the holes and creating a hydrophobic barrier [110]. On the other hand, one can use metal molds, in which the desired pattern is engraved. The paper is placed between two metal molds or between a metal mold and thin glass, followed by immersing it in molten paraffin for a few seconds [112,113]. Accordingly, the metal mold pattern is engraved on the paper, leading to the formation of a hydrophilic substrate. The other parts of the paper are impregnated with paraffin, making the hydrophobic barriers [114]. Although the above-mentioned methods introduce simple and inexpensive processes to fabricate paper sensors, it is not possible to mass-produce them efficiently. One approach to overcoming this limitation is to employ an inkjet printer, engraving the pattern on paper by dropping ink droplets [115]. In this regard, the type of ink, cartridges, and nozzles inside the printer are selected according to the usage type of the device [115]. Polystyrene and alkyl ketene dimers have been used as reagents to create hydrophobic barriers. These reagents are mixed with solvents, such as toluene and heptane [22]. The process of creating a pattern on paper is completed by heating it at high temperatures [116]. One drawback of this process is the use of toxic and environmentally hazardous organic compounds, which can also damage the structure of the printer [22]. In this respect, alternative compounds including acrylate, surfactant, and gel have been used [117]. However, most reagents must be dissolved in volatile solvents, likely clogging the nozzle or causing a non-uniform distribution of the reagent on paper over time.

In addition to the inkjet printing, flexographic printing is also used to mass-produce printing sensors. Unlike the inkjet printing, multiple layers of printing are required in the flexographic printing to create hydrophobic barriers on paper, printing the design continuously on successive rolls [118]. In this case, polystyrene is used as a reagent, which is dissolved in a toluene solvent or xylene group [119]. As well, there is no need to heat the paper to make it hydrophobic [119]. However, flexographic printers are not cost-effective, and their cleaning and preparation processes are complex. The uniformity of the paper surface also affects the print quality [96].

In recent years, commercial laser printers have been used to print designed patterns [120]. The printed paper is placed on a hot plate, at a temperature range of 150–200 °C, in order to perform the hydrophobic process [121]. While the printing limitations are reduced by the laser printers, an additional heating step is required to create hydrophobic barriers [121]. The inks used in these printers are also water soluble. If the hydrophobicity process and penetration into the texture of the paper are not carried out properly, the inks can be dissolved in the injected solution, destroying the pattern created on the surface of the paper. Therefore, the advantages of using laser printers are limited by choosing the appropriate ink [121].

Screen printing is one of the printing-based methods most used to produce electrodes in electrochemical systems [122]. The printing process is carried out with the help of a stencil [122]. The hydrophobic barriers are made by wax, UV-sensitive polymer compounds, polystyrene, TiO_2_ nanoparticles, and conductive paints using different stencils [122]. The working and reference electrodes are positioned in hydrophilic areas. While the fabrication process of the resulting sensors is simple, it is not possible to produce hydrophobic barriers uniformly [123]. Additionally, different patterns need to be designed to perform different electrochemical processes. To create different patterns, different economically unviable stencils must be designed [124]. Screen printing is mainly used to fabricate ion-selective and glucose-sensitive electrodes [124].

Additionally, 3D printers are used to create microfluidic patterns [125]. A layer of ink is scanned on the paper using a computer program. The gaps created are covered with the help of PDMS. UV-sensitive polymers and waxes are employed as inks, allowing for the mass production of paper sensors. Anhydrous alcohol is used to remove the non-hydrophobic substrate [126]. The hydrophilicity of channels and other detection zones increases by modifying the paper surface with compounds such as cellulose [126]. The paper also needs to be heated at a certain temperature [126]. Since the price of a 3D printer is high, it may not be suitable for users.

One of the limitations of the printing methods was their need for space-consuming, expensive printing tools and cartridges, hindering the rapid production of paper sensors ubiquitously. This problem was solved by introducing the stamping method, in which a stamp made of PDMS was immersed in a stable, indelible ink and stamped on paper within less than a few seconds [127]. Unlike previous methods, no modifications were made to remove hydrophobic agents in the hydrophilic channels [127]. In addition to PDMS, stamps can be made of metal and foam materials [128]. Although the resulting stamps are capable of storing inks and easily fabricated under laboratory experimental conditions, their repeated fabrication is not desirable. This leads to the fabrication of different paper-based devices that affect the performance of the sensor.

Spraying is another method that uses no printing process [129]. To carry out the spraying method, the paper is initially covered with a stencil, and the hydrophobic material is then sprayed on its surface [130]. It should be noted that Whatman grade 1 paper is not suitable for this purpose, as it prevents the penetration of the hydrophobic material into the paper texture, thus causing it to leak [130]. Increasing the paper grade from 1 to 4 enhances the possibility of the material penetration into the substrates, thereby creating more hydrophobic barriers [130]. Similar to the stamping method, it is not possible to create microfluidic patterns with high reproducibility, due to the non-uniform spraying.

In the chemical vapor deposition method, a monomeric compound is first vaporized under vacuum conditions to produce radical particles. By polymerizing the particles on the paper surface, together with the help of a mask, hydrophobic barriers are created [131]. The type of the polymers can be poly (chloro-p-xylene), poly (perfluorodecyl acrylate), poly (fluorocarbon), poly (octafluorocyclo butane), and poly (hydroxybutyrate) [132]. There is no need to wash the polymer compound excessively with solvent, unless factors such as metal salts prevent the performance of polymerization [96,133]. One approach to dealing with this issue is to immerse the polymer-impregnated paper in an ethanol bath, thus allowing for the accumulation of the polymer on its surface. The paper is then covered with a mask and exposed to UV light to create channels and hydrophilic zones [134]. However, these methods require a special laboratory equipped with expensive devices [131].

Sensors with 2D structure have been used for different applications because they can be designed in a variety of formats. Some experimental processes require several pre-preparation steps such as separation, preconcentration, filtration, and mixing. The test may also involve the production of gas that must be stored and measured (impossible to perform in a planner condition). A portion of detection reagent can be washed by the sample flow and reduce a part of the sensor response. To solve this problem, the sensor structure needs to be changed from 2D to 3D.

### 5.2. Three-Dimensional Configuration

In 3D structures, the sample is perpendicular to the surface of the paper, passing through various overlapping layers [135]. Each layer is responsible for performing a chemical reaction and transferring the corresponding product to the next layer. The detection element is embedded in the last layer, in order to indicate changes that occurred in the analyte amount [136]. To fabricate a 3D paper device, different methods, including stacking, origami, and double-sided 3D printing, have been employed [23].

**Figure 3:**
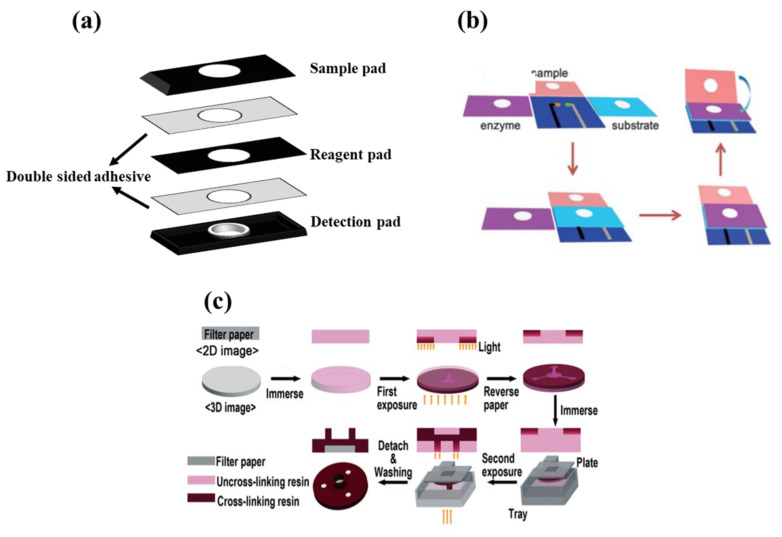
Fabricating methods for three-dimensional configuration. (**a**) Stacking method, (**b**) origami method (reprinted with permission from [140], copyright (2016) John Wiley and Sons), and (**c**) double-sided 3D printing method (reprinted with permission from [139], copyright (2018) The Royal Society of Chemistry).

In the stacking method (Figure 3a), the patterns plotted are implemented on paper using one of the above-mentioned methods of fabricating 2D structures. Different layers of paper are then glued to each other via a double-sided adhesive [93]. The most important limitation of the stacking method is the adjustment of the layers, so that the upper layer patterns match those in the lower layers. The misalignment effects can cause the sample, solvent, and reagent to be wasted, partially resulting in a negative error in the sensor response. Moreover, the adhesives used may even cover some of the hydrophobic areas, preventing the liquid moving [137].

In origami structures (Figure 3b), the flexibility of paper is used to fold different layers created on top of each other. In this respect, hydrophilic zones and hydrophobic barriers are formed using wax printing, ink printing, and screen printing on the paper, separating each layer by a line. The layers are folded over these lines, solving the problems of layer misalignment and hydrophobic area blockage [138].

Finally, double-sided 3D printing (Figure 3c) is a new way of creating 3D structures that has been introduced recently [139]. In this method, the filter paper is exposed to the printer after being modified by resin and PDMS or a light-sensitive polymer, thereby creating hydrophilic areas. This process is repeated for both sides of the paper. The engraved paper is then immersed in an organic solvent (e.g., ethanol), in order to eliminate excess polymeric material from the paper surface, providing a 3D pattern [139]. Accordingly, it is possible to create hydrophilic channels or zones with different widths, lengths, and depths using the double-sided 3D printing method [139].

## 6. Classification of Bio-PPOCT, Based on Bioreceptors

The biosensors use bioreceptors that have the ability to interact specifically with the target analyte, affecting the intrinsic properties of the detection elements [141]. Thus, the ability of a sensor depends on the selectivity of the bioreceptors. A suitable bioreceptor can detect a specific species in a complex matrix, in the presence of interfering species, without needing isolation and purification processes. Nevertheless, the bioreceptor selection depends on the type of analyte, the purpose of the analysis, and the type of transducer used [142,143,144].

### 6.1. Antibody-Based Bioreceptors

The development of immunoassays are based on the interaction of an antibody with a specific compound, such as an antigen [145]. The mechanism of the interaction is based on the lock–key principle, meaning that a particular antigen has an affinity for a corresponding antibody [145]. Hence, these bioreceptors are called affinity bioreceptors and are used to fabricate rapid and serological tests for diagnosing diseases, while also qualitatively detecting contaminants in food and the environment [146]. The interaction between the bioreceptors and the analyte does not lead to an intelligible signal. Therefore, the bioreceptors need to be labeled with a detection element, including nanoparticles, as well as fluorescent, electroactive, and radioactive compounds with the capability of generating the signal [145].

### 6.2. Synthetic Protein-Based Bioreceptors

The affinity constant of the antibody-antigen complex is 10^8^ L per mole, resulting from an irreversible interaction [147]. However, some limitations, such as high molecular weight, low stability of the antibodies at ambient temperature, and the need for special storage conditions, make them difficult to employ in the biosensors. Additionally, their production cost is not affordable [148]. An alternative is to use small synthetic protein structures that can interact with antigens. The affinity constant of the resulting complex is in the range of about 10^2^–10^4^ L per mole, giving rise to a reversible interaction. In other words, one can recover the receptor and reuse it. Unlike antibodies, the protein structures are low in molecular weight, stable in environmental conditions, and inexpensive to produce. Nevertheless, they are mainly used for in vitro techniques [149].

### 6.3. Aptamer-Based Bioreceptors

Another class of bioreceptors is aptamers, consisting of a double-helix structure of nucleotide bases, including adenine, thymine, cytosine, and guanine, that can interact with their complementary bases. Depending on the type of the analyte base, a complementary bioreceptor is selected to induce the hydrogen interaction between adenine/thymine and cytosine/guanine [150]. These structures are called hybrid bioreceptors [151], accurately detecting sensitive and selective microorganisms, such as viruses and bacteria, in the field of spectral detection and food spoilage [152].

### 6.4. Enzyme-Based Bioreceptors

Enzymes are bioreceptors that act as catalysts in a specified reaction; consequently, they are not consumed in the analysis process and can be used for a long time [153]. This feature, along with other advantages, such as the ability of the bioreceptors to pair with different optical and electrochemical transducers, high-detection efficiency of a single or group of analytes, and good compatibility (as a participant in both activation and inhibitory reactions), has made enzymes the most popular receptor in the fabrication of biosensors [154]. The following events occur in enzyme-based reactions: (i) the analyte activates the activity of the enzyme or, conversely, inhibits its performance, and (ii) the analyte is converted to a compound during enzymatic reaction that can be detected by the transducer [155]. Among enzymes, oxidative structures are more commonly used because they are stable compounds that do not require coenzymes. Notwithstanding, a serious problem for these bioreceptors is their reduced activity over time, considerably influencing the sensitivity of the sensor [156].

### 6.5. Microorganisms-Based Bioreceptors

Along with microorganisms, cell organs, and tissues, enzymes fall into the category of catalytic bioreceptors [151]. Organs such as lysosomes, chloroplasts, and mitochondria have been used as receptors [157]. Each of these organs has a different metabolic activity that can be altered by the interaction with the analyte [157]. Microorganisms, such as bacteria, fungi, algae, and yeasts, are receptors that can be easily immobilized on the surface and are resistant to environmental changes. Thus, they can be stable for a long time [158]. Since the microorganisms are so sensitive to changes in the environment, they can be used to detect a wide range of toxic analytes (e.g., herbicides), food spoilage, biological oxygen demand, and even the effectiveness of drugs in treating diseases [159]. The sensor response is obtained based on the amount of analyte absorbed on the receptor or the determination of changes in the receptor’s respiratory performance in a metabolic process [159]. Although the microorganisms are less selective than enzymes, they can be easily recovered by immersion in a nutrient solution.

### 6.6. Tissue-Based Bioreceptors

Tissues are also used to fabricate biosensors. Unlike other receptors, tissues with high environmental stability are more cost-effective and available, making it easier to stabilize them on a solid substrate [160]. Moreover, the tissues do not need to extract or purify enzymes, due to their enzyme-rich environment [161]. However, the tissues do not have good selectivity because they can respond to a wide range of analytes, while also requiring more time to receive the sensor response than other sensors [161].

## 7. Classification of Bio-PPOCT, Based on Immobilization State of Bioreceptors

Since the bioreceptors located on the paper surface are in the path of the sample, part of these materials may dissolve in the sample solution if they are not firmly immobilized on the substrate. Accordingly, the analyte detection process may be disrupted, causing an error in the sensor response. In other words, the sensor practically loses its efficiency. Depending on the type of receptor and analyte, the interaction between them, the detection method, and the sample matrix, the immobilization process of the receptor is carried out by physical (e.g., surface adsorption, entrapment, and cross-linkage) and chemical (e.g., covalent bonding) methods [162,163,164].

The surface adsorption is the simplest method of immobilization, in which the paper substrate is first coated with protein, polymer, and paste layers, followed by placing the receptor on them via van der Waals adsorption forces [164]. BSA, chitosan, polyvinyl alcohol, and polyvinyl pyrrolidone are the most common adsorbents used for this purpose [163]. By the surface adsorption method, the chemical modification of reagents is not required, and recovering the surface is obtained by washing with a suitable solvent [165]. Unfortunately, the resulting sensors do not show good stability against environmental changes such as pH, temperature, and ionic strength of the sample matrix [30].

In the entrapment method, the receptor is trapped inside a microscopic hollow space made of protein, gel, ink, or synthetic polymer, preventing the components from entering or leaving it [166]. In the cross-linkage method, the receptors are interconnected using a double-functionalized reagent, thus forming a carrier-free macroparticle [165]. The resultant macroparticle can have a crystalline or aggregated structure. A high purity receptor is required to form crystalline macroparticles, whereas aggregated species are formed by adding salts, organic solvents, or nonionic polymers to the solution, resulting in the precipitation of bioreceptors [167]. The precipitated species are covalently bonded together. The cross-linkage should not result in blocking the active sites in the receptor structure, avoiding a decrease in the receptor activity and, consequently, in the efficiency of the sensor [167].

Chemical adsorption is performed by establishing a covalent bond between the functional groups present in the receptor structure (i.e., those that do not interact with the analyte) and the active sites at the substrate surface [168]. The surface of the paper is modified by organic polar solvents, such as ethanol [169]. The hydroxyl groups in the substrate bind to the amino, carboxylic, thiol, or hydroxyl groups present in the receptor structure [169]. The chemical adsorption method has better stability than physical methods, leading to the highest immobilization and lowest receptor leakage.

## 8. Classification of Bio-PPOCT, Based on Detection Elements

The sensor must be able to provide an intelligible signal after the interaction of analyte and bioreceptor. In this regard, detection elements with unique optical, electrical, thermal, and acoustic properties need to be immobilized separately on a hydrophilic zone of the paper reacting with the analyte-bioreceptor reaction product. Sometimes, the bioreceptor is physically or chemically coated by the detection element; thus, changes in the reaction caused variations in the structural, spatial, and environmental characteristics of the receptor. Consequently, the physiochemical properties of the detection element change as well. Detection elements fall into different groups, such as organic dyes, inorganic dyes, and nanoparticles [59]. Organic dyes include redox indicators, pH-sensitive detectors, and chemo-responsive dyes [44].

### 8.1. Redox Indicator

The redox indicators cause significant electrical or color changes, due to the conversion of the reduced form to the oxidized one [170]. These indicators must be able to generate a rapid and reversible response, establishing a rapid chemical equilibrium between the reduced and oxidized forms [171]. Additionally, electro-optical properties of the redox indicators should not be affected by those of other sensor components, such as the receptors, amplifiers, inhibitors, etc. [59]. Given the above-mentioned conditions, only a limited number of organic compounds (e.g., potassium iodide (KI) [172], 2,2′-azino-bis (3-ethylbenzothiazoline-6-sulphonic acid) (ABTS) [173], 3,3′-5,5′tetramethylbenzidine (TMB) [174], 3,3′diaminobenzidine (DAB) [175], o-phenylenediamine (OPD) [176], thionine [177], methylene blue [178], and indigo carmine [179]) can be used for this purpose.

### 8.2. The pH-Sensitive Indicators

In some cases, the interaction between analyte and bioreceptors leads to a change in the acidic and alkaline conditions of the reaction medium, which can be monitored using pH-sensitive indicators [180]. They are weak organic acids and bases whose color changes in the test solution depend on the proton (H_3_O^+^) concentration [181]. The most popular paper sensor (containing pH indicators) is litmus paper, which is made of 7-hydroxyphenoxazone [182]. Sometimes the reaction leads to a change in the physical or chemical nature of the reaction medium, including temperature, solvent polarity, and electrophilic or nucleophilic interactions. Thereby, chemo-responsive dyes can be used depending on the type of the reaction [183]. These dyes are in the categories of Lewis acids and bases, as well as solvatochromics or thermochromics [184]. 

### 8.3. Inorganic Complexes

Inorganic complexes that are a combination of a transition metal and a ligand can also be used as a detection element [185]. In this case, the product of the analyte-receptor reaction with the complex gives an alternative or a combinatory reaction. In the substitution reaction, the product replaces the ligand of the complex composition, which is known as the indicator displacement method (IDM) [185]. To have this mechanism, the metal-ligand complex formation constant should be less than that of the metal product [186]. In a combinatory reaction, the product interacts with the complex to form a ternary structure [135]. The ternary structure is induced by d–d interactions between the product and the metal, or by electrostatic, hydrogen, covalent, and charge transfer interactions between the product and the ligand [135]. Both alternative and combinatory reactions change the electro-optical properties of the initial complex. It is worth noting that the selectivity and sensitivity of the sensor in the presence of inorganic complexes are higher than organic indicators [185].

### 8.4. Nanopaticles

The other category is nanoparticles, having different physical, optical, electrical, catalytic, and biological properties than their bulk counterparts [187]. This arises from the small size of nanoparticles (ranged between 10 and 100 nm) and the dominance of surface forces over volumetric ones [187]. The properties of nanoparticles depend on the type of central metal, reducing and coating agents, size, shape, surface electrical charge, and their distribution state [188]. So far, a wide range of metallic and non-metallic nanoparticles have been used in analytical studies, among which gold, silver, copper, palladium, platinum, carbon, cobalt, and metal oxide nanoparticles have been employed in the fabrication of biosensors [189]. Gold, silver, and copper nanoparticles have shown better optical properties than other nanoparticles, arising from their surface plasmon resonance effect [8]. In fact, a high-intensity absorption band has been observed in the UV-visible region for the nanoparticles, due to the surface plasmon resonance, giving rise to a higher (up to 10^4^ times) molar absorption coefficient than organic indicators [8]. Meanwhile, gold, platinum, and palladium nanoparticles have been employed in the fabrication of electrochemical biosensors, due to their high electrochemical properties and conductivity [190].

Various chemical compounds are used as reducing, coating, and preserving agents in the synthesis of nanoparticles. Some of these compounds are toxic and carcinogenic, limiting their biological and medical applications. Nowadays, the use of green methods is strongly preferred, giving rise to the production of environmentally friendly compounds [191]. In this regard, actinomycetes, bacteria, fungi, yeasts, tissues, and plant extracts have been employed as green reducing agents [191]. The synthesis of chemical compounds using plant extracts is more cost-effective and faster than other reducing agents, avoiding the involvement of isolation, purification, preparation, and maintenance of the culture medium [191]. As a result, large volumes of nanoparticles can be synthesized based on the green methods [191].

### 8.5. Bimetallic Nanoparticles

Nanoparticles can be combined to form a bimetallic nanoparticle, possessing new physicochemical properties, in addition to those found in single metal nanoparticles [192]. Therefore, the figures of merit of the resulting sensor (such as sensitivity, selectivity, and linear amplitude) are enhanced [192]. Most bimetallic nanoparticles fall into the following two categories: core-shell and alloy structures [193]. In the former, a metal is initially reduced to form a core, followed by the precipitation of the second metal on the primary core. This forms a thin layer called the shell [193]. In the latter, both metals are reduced simultaneously using a reducing agent, inducing an intertwined structure. In this way, the properties of alloy nanoparticles change, by varying percentages of metal cores [193].

### 8.6. Magnetic Nanoparticles

Magnetic nanoparticles use a magnetic substance as the core and a chemical compound as a reducing or coating agent [194]. Generally, the metal cores are composed of iron, nickel, and cobalt [195]. The magnetism of the particles is activated in the presence of an external field and it is lost by removing the magnet [194]. Sensors fabricated based on the magnetic nanoparticles are used in a variety of biological applications, as well as in the detection of environmental pollutants [196]. Depending on whether the biological study is carried out inside or outside the body, the use of magnetic nanoparticles can be variable [196]. Notably, the nanoparticles are used to treat (e.g., tracking a drug) or diagnose a disease in the in vivo conditions, whereas they isolate a specific species from the sample matrix or catalyze a chemical reaction in the in vitro conditions [196].

### 8.7. Carbon Nanoparticles

Alternatively, the use of carbon nanoparticles in the fabrication of biosensors, drug tracking, cancer diagnosis and treatment, and imaging the inside of the body have been reported in numerous articles [197]. The carbon-nanoparticle-based biosensors are often coupled to electrochemical transducers [197]. The nanoscale carbon is synthesized in the form of nanotubes, graphene oxide, graphene quantum dots, and fullerene [198]. One of the carbon structures is graphene, consisting of a network of sp^2^ hybrid carbon, in the form of a flat plate [199]. The length of the carbon–carbon bond in the graphene structure is 1.42 Å [199]. Furthermore, the high surface-to-volume ratio of graphene structures, along with the ability to modify their surface with different functional groups, makes it possible to detect a wide range of compounds in extremely low concentrations with high selectivity [199]. Graphene monolayers can be placed on top of each other with the help of van der Waals forces, forming a 3D graphite structure. The gap between the layers is 3.42 Å [200]. By rotating the graphene layer around its axis, hollow cylinders (so-called carbon nanotubes) are created [201]. If a cylinder is made of a high-grade, single-layer graphite sheet, it is called a single-walled nanotube. On the other hand, a multi-walled nanotube is made of several sheets of graphite rolled together [202]. In this case, the distance between the graphite sheets, relative to each other, is 3.4 Å [202]. The multi-walled nanotubes outperform the single-walled ones, in terms of electrical conductivity and mechanical stability. Additionally, the multi-walled nanotubes can have various electrical and structural behaviors for different applications [202]. Fullerene is another form of the carbon structure created by the spherical rotation of graphene layers [197]. Depending on the number of layers, graphene can be divided into thin and thick categories, possessing different electrical, optical, and mechanical properties. To form graphene quantum dots, the layers with lateral dimensions of less than 100 nm are placed side by side, leading to good physical and chemical stability, low toxicity, and high photoluminescence emission of graphene, which can be used in a wide range of wavelengths from infrared to UV [203]. As a detection element, carbon structures have good conductivity and electron transfer rate, allowing for the suitable immobilization of bioreceptors on their surface. In turn, this increases the sensor performance [197].

### 8.8. Nanoclusters

Nanoclusters are particles with sizes less than 5 nm, having different physicochemical properties, compared to nanoparticles [204]. Moreover, nanoclusters do not show plasmonic properties, behaving like a molecule (in the range between atoms and nanoparticles), in which electron structures are discrete, due to the proximity of nanoclusters to the Fermi wavelength of metals. Thus, the enhanced magnetic properties, conductivity, and luminescence of nanoclusters are achieved [205]. The emission intensity of the nanoclusters can also be changed by controlling their size or by selecting an appropriate coating agent, giving rise to good optical stability and biocompatibility [205]. The advantage of nanoclusters, over organic fluorophores, is that they do not suffer from photobleaching. Additionally, the toxicity and physical size of nanoclusters are less than those of quantum dots, allowing for their use in in vivo studies [204].

## 9. Classification of Bio-PPOCT, Based on the Detection Method

The interaction between the analyte and bioreceptor in the sensor structure leads to changes in the optical, electrical, and thermal properties of the detection element, which can be converted into an intelligible signal by a transducer.

### 9.1. Electrochemical Transducer

If the analyte detection is associated with the production or consumption of electrons, an electrochemical transducer will be used, involving working, reference, and auxiliary electrodes [206]. The analyte is detected on the surface of the working electrode, enabling the rapid detection of low-risk species. It is possible to increase the surface and conductivity of the electrode by modifying it, thus improving and amplifying the signal [206]. In some cases, the reaction of an electroactive species is carried out at a certain potential, while also measuring the current produced (i.e., the amperometric method). In fact, the current changes are proportional to the concentration of the species in the sample [206]. Amperometric sensors are classified into three categories: (i) sensors that monitor the amount of oxygen consumed in a reaction using a Clark oxygen electrode; (ii) sensors that use a redox intermediate to transfer electrons between the bioreceptor and the electrode; and (iii) sensors that directly transfer electrons (acting as a catalyst) between the bioreceptor and the electrode [207]. An example of a sensor of the first category is one to determine glucose using an enzyme system (glucose oxidase) [207]. In order to determine the current, cyclic voltammetry, normal pulse voltammetry, and differential pulse voltammetry, methods have been used. In potentiometric sensors, performing a chemical reaction leads to a change in the potential, involving the electrode surface with high sensitivity to the desired species or environmental conditions, such as pH [208]. The changes in the electrode potential are measured against the potential of a reference electrode, pertaining to the logarithm of the concentration [208]. It is possible to place an amplifier in the sensor structure to amplify the resulting signal [208]. The sensitivity, accuracy, and speed of measurement of the potentiometric methods are less than amperometric techniques [206]. The measurements can be carried out by observing the resistance vs. current behavior in the circuit, which changes over time [209]. To this end, a bridge circuit with three electrical resistors (having two known resistors and one variable resistor) is used to calculate the unknown resistance. The variable resistance value is adjusted so that the potential difference in the circuit becomes zero, eliminating nonspecific changes in the unknown resistance, due to variations in the temperature, concentration of dissolved gases, and evaporation. Impedance-based biosensors are mostly used to detect pathogens, such as bacteria, in food or biological samples [209]. Chemical reactions can give rise to the production of a charged species, thus changing the conductivity of the solution, measured by a conductometer. Since these sensors do not have good selectivity and sensitivity, they are used less, compared to other electrochemical sensors [210]. Another applicable method that has attracted the attention of researchers nowadays is the electroluminescence method, exciting the target species by an electrochemical stimulus [211]. Unlike the photoluminescence method, the interference caused by light scattering and luminescence background in the electroluminescence method is minimized, thereby increasing the signal-to-noise ratio. In turn, this enhances the sensitivity, accuracy, and precision of the method [211]. In other words, the electroluminescence method has good reproducibility and can be used in concurrency analysis. Moreover, the combination of the electroluminescence transducer with microfluidic structures enables us to determine the sample with an extremely small volume (1.8 microliter) and a low detection limit (~1.5 femtomoles) [212].

Among electrochemical methods, potentiometric and amperometric ones have received more attention from researchers. These methods are more useful for fabricating the point-of-care sensors. The most common electrochemical biosensors are the glucometer [213] and alcohol breath analyzer [214]. A drop of blood or a few seconds of exhalation is sufficient to cause a reaction. However, the accumulation of biological species on the electrode surface can reduce its transfer rate, leading to a negative effect on the sensor performance [215]. Today, electrochemical sensors are connected to a wireless control system, enabling us to monitor electroactive species remotely, while also transferring data from one device to another via Bluetooth [216].

### 9.2. Thermal Transducers

In thermal transducers, the changes in the heat produced or consumed in a chemical reaction are measured over a period of time [157]. Depending on the temperature range and the importance of the sensor resistance in physical and chemical environments, different thermal transducers can be used [217]. Notably, thermocouples are the most robust thermal sensors; they have good physical and chemical stability and operate in the temperature range of −200 °C to +3000 °C [218]. On the other hand, thermistors and semiconductors are employed in the temperature ranges of −50 °C to +300 °C and −40 °C to +100 °C, respectively [218]. These sensors have lower thermal resistance and stability than thermocouples [218]. Sometimes the thermal sensor information is not accurate and reliable, arising from factors such as heat loss (due to radiation or convection), sensor heating (after applying an electric field generated from an external power supply), and sensor deformation (due to mechanical pressure). Accordingly, thermal biosensors have rarely been considered in research studies [151].

### 9.3. Optical Transducers

Optical transducers are very popular among all transducers employed in the preparation of paper sensors because of their simplicity, availability, and capability [17]. The analyte-bioreceptor interaction leads to a change in the optical property of the detection element, which can be observed in the form of absorption, emission, scattering, and reflection of light. Depending on the type of change, colorimetric, and fluorometric methods can be used [17].

In the colorimetric method, changes taking place in the intensity of light absorbed or reflected are observed [219]. In fact, chromophores with different configurations cause the optical property to change after the reaction. In the case of nanoparticles, the change in the optical property arises from their aggregation or surface modification [219]. These changes are proportional to the amount of analyte in the sample. The most important advantage of the colorimetric methods is that the color changes can be seen with the naked eye [219]. It is also possible to obtain more accurate detection results by performing image analysis or spectrophotometric spectrum analysis. The absorption spectrum of the detection element is investigated before and after the interaction using the spectrophotometry analysis. Basically, the maximum wavelength is used for quantitative measurements [8]. The changes can decrease or increase the absorption at specified wavelength or can shift the peak to lower or higher wavelengths [8]. Scanners, cameras, or smartphones are used in the image analysis [220]. In this respect, the image of the detection element is obtained before and after the color changes, followed by calculating the values of the color (red, green, and blue) components for each image, pixel by pixel. The average values obtained for each color component are then calculated, and the difference between these values is determined before and after the interaction. The numerical average values are returned to the image format, creating a color-difference pattern [28]. The intensity of the resultant color difference is proportional to the concentration of analyte in the sample. Alternatively, the image analysis can certainly be a simpler and more appropriate approach for the development of a point-of-care method. In this case, the white background of the paper substrates does not interfere with the measurements [220]. However, the optical conditions of the medium can interfere with the detection of color changes. For this reason, the image analysis is performed under a controlled light condition, in a sealed cabinet [221]. The main problem of the image-analysis-based methods is their low sensitivity and long response time. Moreover, it might be necessary to use a signal processor to achieve a desired signal, thus increasing the time and cost of the analysis.

In the fluorometric method, the return of the excited species to the ground state results in the emission of photons with less energy and longer wavelengths [222]. Different fluorophore compounds, such as chemical dyes, semiconductor quantum dots, carbon quantum dots, and nanoclusters can be employed as detection elements in the fluorometric sensors [223]. The reaction between the analyte and the bioreceptor may increase or decrease the fluorescence intensity, which can be attributed to the effect of internal filtering, dynamic damping, static damping, or Förster resonance energy transfer (FRET) [224]. Unlike colorimetric methods, the fluorometric sensors are highly selective and sensitive when employing a fluorometer or a fluorescence-sensitive camera [225]. The sensor is placed on a UV lamp with an excitation wavelength of 365 nm, monitoring the fluorescence emission via the camera [225]. In these methods, the fluorescence of the paper substrate interferes with the measurement, which can be removed by subtracting the sample fluorescence from the background fluorescence, by using a standard compound (thus verifying the measurement validity), or by embedding a filter in the signal receiving device [165].

## 10. Application of Paper Biosensors in the Detection of Toxic Materials

In continuance, we review the studies carried out on the development of paper biosensors in the detection of mycotoxins, organophosphates, bacteria, and metal ions. Of course, the instrumental and analytical information of these biosensors are summarized in Table 1, Table 2, Table 3 and Table 4.

### 10.1. Mycotoxins Detection

Toxins can be of biological origin, such as plants, animals, bacteria, and fungi. According to the source of production, toxins can be classified into various categories, such as botulinum neurotoxin, conotoxins, diphtheria toxin, notexin, tetrodotoxin, phycotoxins, phytotoxins, and so on [226]. Among them, mycotoxins are secondary metabolites produced by fungi, such as *Fusarium*, *Aspergillus*, and *Penicillium*, when harvested or stored improperly [227]. These toxins can enter the human body either directly (through the consumption of contaminated agricultural materials) or indirectly (through the consumption of animal products), causing cancer in tissues, as well as gene, liver, and kidney poisoning [228]. Moreover, they may cause disorders in the nervous and reproductive systems [228]. So far, a wide range of mycotoxins have been identified. The best known of these are: aflatoxins (AF), ochratoxins (OTA), fumonisins, patulin, zearalenone (ZEA), and trichothecenes [229]. According to the International Agency for Research on Cancer (IARC), mycotoxins can be divided into two categories: potent carcinogens (e.g., aflatoxins) and substances that can be (but are not necessarily) carcinogenic (e.g., ochratoxins) [230].

Among mycotoxins, many studies have been carried out on aflatoxins [231]. In 1960, aflatoxin was found to be the cause of the turkey X disease [232]. This toxin is a metabolite produced by the growth of *Aspergillus flavus* and *Aspergillus parasiticus* [231]. These fungi can be found in agricultural products, such as wheat, peanuts, bran, sesame seeds, peppers, and a variety of spices [233]. However, the growth of fungi increases by keeping these species in certain conditions, such as humidity above 7% and temperatures between 13 °C and 40 °C [234]. According to the Food and Drug Administration (FDA), the permissible level of aflatoxin in food samples should be between 20 and 300 ppb [235]. The aflatoxins can be classified as aflatoxins (AF) B1, B2, G1, G2, M1, and M2 [230]. Among them, the most toxic type of aflatoxins is aflatoxin B1, resulting in poisoning through both swallowing and skin penetration [236]. The liver, the most important organ in the body, is severely affected by aflatoxin B1 [236]. Liver failure causes fatty infiltration, necrosis, hemorrhage, fibrosis, regeneration of nodules, and even bile duct proliferation/hyperplasia [236]. To prevent these problems, the presence of aflatoxin B1 and other types of mycotoxins should be detected, and its amount be determined using analytical methods. Traditional methods, such as chromatography (e.g., thin layer and high performance liquid chromatography), spectrophotometric methods (e.g., colorimetry or fluorimetry), and enzyme-linked immunoassay methods, are used for the qualitative and quantitative analysis of aflatoxins [227]. While these methods are accurate and sensitive, they are costly and time-consuming and require special laboratory conditions. Certainly, using a biosensor that can detect and measure the toxins at the sampling site, with the help of a dedicated receptor, could be a simpler and more user-friendly method.

As seen in Table 1, most biosensors employed to detect and measure mycotoxins have a 2D design and lateral flow structure. In research studies, mycotoxins are extracted from agricultural and food samples. The bioreceptor of the resulting sensors is primarily a monoclonal antibody. The antibodies are either conjugated to gold nanoparticles with a colorimetric transducer or coated on a fluorophore, in which the amount of mycotoxin is detected by fluorescence. The reasoning behind the use of a fluorescence probe is that, since the substrate is covered with a black background, due to the color of the analyte (thus being unsuitable for the colorimetric method), it does not properly show the changes in the color of the gold nanoparticles [237].

**Figure 4:**
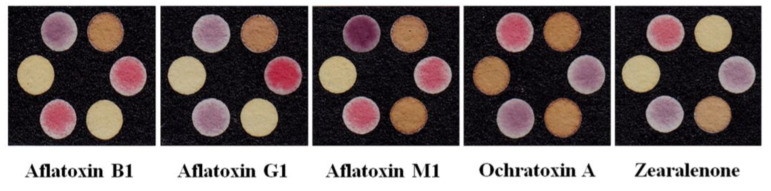
Colorimetric sensor array, based on gold and silver nanoparticles for the individual and simultaneous detection of alfatoxins and ochratoxin A, zearalenone (reprinted with permission from [250], copyright (2020) Springer Nature).

Most lateral flow-based methods use a competitive mechanism for the interaction between the antigen and antibody in the test line. This approach was used for the detection of AFB1, by using a 1-ethyl-3(3-dimethylaminopropyl) carbodiimide hydrochloride (EDC)-mediated method [237] or AFM1 with polystyrene microspheres, enclosing time-resolved fluorescent europium (III) [Eu(III)-TRFM] [238]. The TRFM immunoassay is a new method, based on lateral flow, in which fluorescence microspheres are used as probes, giving rise to good sensitivity and a high linear range [239]. Most TRFM lanthanide complexes are used to make these sensors. Lanthanide elements, such as Eu(III), Tb(III), Sm(III), and Dy(III), are involved in the formation of the complexes [238]. The fluorescence intensity of the resultant compounds is weak, reducing the sensor’s performance in detecting extremely small amounts of analyte. Tang and Wang et al. proved, in two separate studies, that the fluorescence emission intensity of the complexes could be increased by encapsulating them in polystyrene or by chelating them with silica nanoparticles, respectively [240,241]. In a typical study, Tang et al. [242] used this strategy for detection of six types of hazardous chemical compounds, including AFB1, AFB2, AFG1, AFG2, carbaryl, and carbofuran. These compounds were measured for five corn samples, with detection limits of 0.03 ng·mL^−1^, 0.02 ng·mL^−1^, and 60.2 ng·mL^−1^ [242]. In lateral flow colorimetric methods, the detection element is mostly made of gold nanoparticles (mainly synthesized by sodium citrate). Unlike fluorescence, these methods do not interfere with the fluorescence emission of the substrate. The detection is also possible with the naked eye and does not require an excitatory stimulus, such as the UV lamp. Extensive studies have been performed based on lateral flow colorimetric methods. Notably, according to Wang et al. [243], the colorimetric method shows good performance in detecting AFM1 in raw milk samples in the presence of microorganisms, such as *Escherichia coli* O157:H7, having a detection limit of 50 pgmL^−1^. In this regard, two test lines, one for the AFM1 competitive reaction and the other for the detection of *Escherichia coli*, were embedded in the detection pad [243]. This design has also been observed in the simultaneous detection of different species of mycotoxins. In fact, a wide range of mycotoxins exist in real samples. Instead of fabricating a separate sensor for each mycotoxin, it is possible to place bioreceptors of all toxins on a pad, thus reducing the cost and time of analysis. In simultaneous measurements, coating antigens of different toxins (e.g., aflatoxins, ochratoxins, deoxynivalenol, ochratoxin A, T-2 toxin, and zearalenone) are sprayed along each other on the detection pad [68,244,245]. The detection limits of these sensors are given in Table 1. As can be inferred, most of the sensors used in the simultaneous detection have the same design. Nevertheless, the visual detection limits for determining the amount of a particular toxin, obtained by the various methods, are different from each other because the detection and determination of toxins depends on factors such as the antibody concentration, nanoparticle concentration, nanoparticle size, pH value of Ab-GNP interaction, blocking buffer, and the order of the test lines [68]. For example, the use of BSA in the blocking buffer composition is suitable for detecting AFB1, whereas it is not effective for detecting OTA. This is due to the interaction of BSA with OTA, thereby reducing the sensitivity of the measurement. Therefore, ovalbumin (OVA) is used in the blocking buffer of a mixture containing AFB1 and OTA [246]. The pH, antibody concentration, and nanoparticle concentration should be such that a stable Ab-GNP conjugate is formed, avoiding the aggregation of nanoparticles [68]. The Ab-GNP conjugate instability can affect the formation of a stable test line, thus changing the sensitivity and performance of the sensor [68]. Basically, pH affects the electrostatic interaction between nanoparticles and antibodies, so that the antibody creates a large steric hindrance, preventing the nanoparticles from being aggregated [68]. On the other hand, experiments have shown that the greater the distance between the test line and the conjugate pad, the better the measurement sensitivity [68]. Of course, this is a relative phenomenon, which can be suitable for some toxins and not effective for other toxin types. In addition to antibodies, aptamer can be used as bioreceptor in lateral flow structure. This system was used by Zhang et al. [247] for the detection of OTA in corn samples through fluorescence techniques. Limited studies have been performed using microfluidic structures, likely due to the type of paper. In fact, it is difficult to fix the bioreceptor on the Whatman paper used in the fabrication of microfluidic devices. This contrasts with lateral flow sensors, employing a nitrocellulose paper as the substrate. However, these tools have been used to detect mycotoxins, establishing the detection mechanism, based on the interaction between toxins and aptamer. The aptamer of a specific toxin is initially absorbed on a gold nanoparticle and then separated from it by adding analyte to the solution. The high affinity of the aptamer for the analyte is responsible for this separation, followed by the interaction with the toxin. Moreover, the configuration of the aptamer changes to G-quadruplex [248]. If a salt, such as NaCl, is added to the solution, the charge repulsion between the nanoparticles is reduced, causing them to accumulate and change color from red to blue [248]. Kasoju et al. have employed this strategy to determine AFB1 and AFM1 in food and milk samples, respectively. Thus, the concentration of analytes can be determined with an accuracy of picomolar [249]. To perform simultaneous measurements in microfluidic structures, Sheini [250] proposed a nanoparticle-based color sensor array, without the use of bioreceptors. In this case, the interaction between the analyte and the organic compounds, coated on silver and gold nanoparticles, was responsible for the color changes. In fact, the accumulation of nanoparticles in the presence of analytes gives rise to the color changes. As shown in Figure 4, the sensor array creates a unique pattern for each toxin that can be used to determine the type of toxin (and its chemical structure) and to produce fungus, leading to a detection limit in the range of nanomolar [250]. Mycotoxins have also been evaluated by electrochemical methods. In this respect, Migliorini et al. have been able to detect AFB1 by an electrochemical sensor, in which the anti-AFB1 antibody was covalently immobilized on a film prepared by multi-walled carbon nanotubes and chitosan. The resulting sensor provided an AFB1 detection limit of 0.62 ng·mL^−1^ and excellent recovery in the corn sample [251].

**Table 1:** Different type of paper-based biosensor for mycotoxin detection.

Type of Mycotoxin	Device Structure	Bioreceptor	Detection Method	Sensing Element	Media	Linear Range	Detection Limit	Ref.
AFB1	Immunofiltration assay	Anti-AFB1	Colorimetric	AuNPs	Rice, corn, and wheat	0–4000 ng·mL^−1^	2.0 ng·mL^−1^	[252]
AFB1	Immunodipstick assay	Anti-AFB1	Colorimetric	Core-Shell AgAuNPs	Rice, wheat, sunflower, cotton, chillies, and almonds	0.1–10.0 ng·mL^−1^	0.1 ng·mL^−1^	[253]
AFB1	Microfluidic assay	Aptamer	Colorimetric	AuNPs	-	1 pM–1 mM	10 nM	[254]
AFB1	Lateral flow assay	Anti-AFB1	Fluorimetric	Fluorescent microsphere	Soybean sauce	-	2.5 μgL^−1^	[237]
AFB1	Printed electrode	Anti-AFB1	Impedimetric	MWCNT/chitosan	Maize flour	1.0 to 30.0 ng·mL^−1^	0.62 ng·mL^−1^	[251]
AFM1	Lateral flow assay	Anti-AFM1	Colorimetric	AuNPs	Milk	-	50 pgmL^−1^	[243]
AFM1	Microfluidic assay	Aptamer	Colorimetric	AuNPs	Milk	1.0 pM to 1.0 µM	10.0 nM	[249]
AFM1	Lateral flow assay	Anti-AFB1	Fluorimetric	Fluorescent microsphere	Milk powder, UHT, and pasteurized milk	0.05–2.0 ng·mL^−1^	0.019 ng·mL^−1^	[238]
OTA	Lateral flow assay	Aptamer	Fluorimetric	Fluorescent probe	Corn	1–1000 ng·mL^−1^	0.40 ng·mL^−1^	[247]
AFs	Lateral flow assay	Anti-AFs	Fluorimetric	Eu(III) NPs	Corn	0.03–3.90 ngg^−1^	0.03 ngg^−1^	[242]
AFB1, AFM1, DON, OTA, T-2, ZEN	Lateral flow assay	Anti-AFB1, Anti-AFM1, Anti-DON, Anti-OTA, Anti-T-2, Anti-ZEN	Fluorimetric	Protein microarrays	Water	0.04–1.69 ng·mL^−1^ 0.45–3.90 ng·mL^−1^ 20.20–69.23 ng·mL^−1^ 35.68–363.18 ng·mL^−1^ 0.11–1.81 ng·mL^−1^ 0.08–7.47 ng·mL^−1^	0.01 ng·mL^−1^ 0.24 ng·mL^−1^ 15.45 ng·mL^−1^ 15.39 ng·mL^−1^ 0.05 ng·mL^−1^ 0.01 ng·mL^−1^	[246]
AFB1, OTA, ZEN	Lateral flow assay	Anti-AFB1, Anti-OTA, Anti-ZEN	Colorimetric	AuNPs	Peanuts, maize, and rice		0.25 ng·mL^−1^ 0.5 ng·mL^−1^ 1.0 ng·mL^−1^	[244]
AFB1, OTA, ZEN	Lateral flow assay	Anti-AFB1, Anti-OTA, Anti-ZEN	Colorimetric	AuNPs	Corn, rice and peanut		0.10–0.13 μg·kg^−1^ 0.42–0.46 μg·kg^−1^ 0.19–0.24 μg·kg^−1^	[68]
ZEAs, DONs, T-2s, AFs, FBs	Lateral flow assay	Anti-ZEAs, Anti-DONs, Anti-T-2s Anti-AFs, Anti-FBs,	Colorimetric	AuNPs	Cereal		0.04–0.17 μg·kg^−1^ 0.06–49 μg·kg^−1^ 0.15–0.22 μg·kg^−1^ 0.056–0.49 μg·kg^−1^ 0.53–1.05 μg·kg^−1^	[245]
AFB1 ZEA DON	Lateral flow assay	Anti-AFB1, Anti-ZEA, Anti-DON,	Colorimetric	AuNPs	Cereals		0.05 μg·kg^−1^ 1.0 μg·kg^−1^ 3.0 μg·kg^−1^	[255]
AFB1 AFG1 AFM1 OTA ZEN	Microfluidic assay	Free	Colorimetric	AuNPs and AgNPs	Pistachio, wheat, coffee, and milk	3.1–7800 ng·mL^−1^ 8.2–8400 ng·mL^−1^ 2.5–8200 ng·mL^−1^ 4.0–3800 ng·mL^−1^ 8.0–7900 ng·mL^−1^	2.7 ng·mL^−1^ 7.3 ng·mL^−1^ 2.1 ng·mL^−1^ 3.3 ng·mL^−1^ 7.0 ng·mL^−1^	[250]

AF: aflatoxin; NP: nanoparticle; MWCNT: multi-walled carbon nanotubes; UHT: ultrahigh-temperature; OTA: ochratoxin A; fluorescent probe: 5′-biotin-(CH2)_6_-ttt-ttt-ttt-ttt-ttt-ttt-3′; DON: deoxynivalenol; T-2: T-2 toxin; ZEN: zearalenone; FB: fumonisin.

### 10.2. Organophosphates Detection

**Figure 5:**
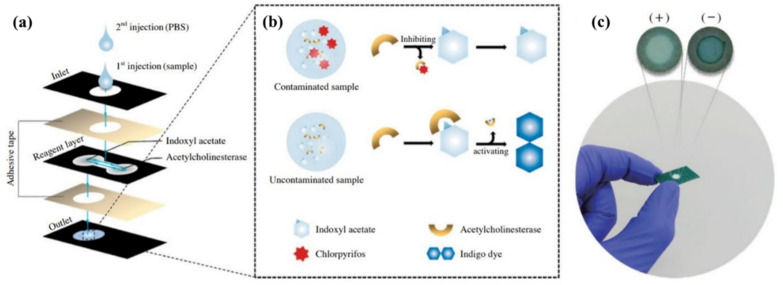
3D sensor configuration, obtained via the stacking method. The sensor was used to detect chlorpyrifos through the enzymatic procedure. (**a**) The proposed procedure for creation of sensor and detection of pesticide; (**b**) the proposed mechanism for detection of pesticide: ACHE and indoxyl acetate enzymes reacting together to produce indigo blue (the intensity of blue color was reduced after the entrance of the sample from the first layer and inhibition of enzyme activity); and (**c**) the response of the sensor in the presence of contaminated (positive) and normal (negative) samples (reprinted with permission from [262], copyright (2018) Springer Nature).

Pesticides are divided into three categories: organochlorines, organophosphates, and carbamates [215,256]. Among them, the tendency to produce organophosphates is higher, as their decomposition process is rapidly carried out, with the help of microorganisms or natural environmental processes [7]. However, organophosphates have a long half-life and accumulate in the environment for a certain period of time. Other types of organophosphates can act as nerve agents, which are used in chemical warfare, or enter into the river and groundwater cycle from the waste of industrial products, such as plastics, lubricants, refrigerants, fuels, solvents, dispersants, and surfactants [7,257]. The chemical structure of these compounds falls into the two following groups: oxon (P=O) and thion (P=S) [258]. Both groups have the ability to inhibit the activity of cholinesterase enzymes that act as neurotransmitters, giving rise to the acetylcholine accumulation in the intersynaptic space [258]. In turn, this results in neurological, respiratory and cardiovascular disorders and even death (in acute cases) [258]. The organophosphate toxicity, which has been proven since 1960 [259], poisons 3 million and kills 200,000 people annually [260]. Many efforts have been devoted to detecting and determining these compounds (quickly and in a timely manner) in real samples, one of which is the use of biosensors. As represented in Table 2, in organophosphate biosensors, most enzymes are used as bioreceptors, involving the mechanism of enzyme activity inhibition by organophosphate in the body. In this mechanism, acetylcholine is initially hydrolyzed to choline in the presence of acetylcholinesterase. The resultant product can react with the detection element (being made of gold or silver nanoparticles) used in the sensor structure, thus changing its color. In another mechanism, the choline produced in the presence of choline oxidase is oxidized to H_2_O_2_, followed by its participation in the oxidation reaction with a redox organic substance that changes the oxidized state of the colored substrate to a reduced form, resulting in a change in its color [261]. The colorimetric reagent used so far for these sensor systems are indophenol acetate, dithiobisnitrobenzoate, indoxyl acetate, and 3,3′,5,5′-tetramethylbenzidine. In one study, a three-layer sensor was designed to detect chlorpyrifos [262]. The configuration, mechanism of reaction, and response of the sensor are shown in Figure 5. Using double-sided adhesive, the layers were stacked on top of each other, so that the middle layer contained the ACHE and indoxyl acetate reacting together to produce indigo blue. The production process of indigo and, subsequently, the intensity of the blue color was reduced after the entrance of the analyte from the first layer and the inhibition of enzyme activity. The resulting color changes could be seen in the third layer [262].

The origami method can also replace the stacking one, due to its easier and more accurate fabrication process of a 3D system. By considering a bilayer sensor, comprising of the ACHE enzyme in the first layer and indophenol acetate in the second layer, the solution sample is injected into the enzyme-containing layer [263]. After folding the layers, the interaction between the enzyme and the colorimetric indicator occurred, and the color of the sensor changed from blue to colorless in the presence of phosphorus analytes (e.g., chlorpyrifos). The possibility of tracking the analyte amount in the sample using any type of smartphone has been proven, as well. In this way, ambient light sensors were used and installed on smartphones [263]. In these sensors, it is possible to control the ambient light conditions and adjust the backlight, preventing the sensitivity of the CMOS sensors or the quality of the camera lens from affecting the reception of high-resolution images [263]. The color indicators can be replaced by nanoparticles, whose central metal atom is capable of participating in the oxidation reaction [264]. The most common type of nanoparticles is the metal oxide, including nanoceria (CeO_2_) nanoparticles [265]. The interaction of these nanoparticles with H_2_O_2_ changes the Ce^3+^/Ce^4+^ ratio on the surface, thus varying their color from colorless to yellow [266]. In the presence of organophosphates, the activity of enzyme was inhibited thereby reducing the H_2_O_2_ amount produced, and turning the paper color from yellow to colorless [267]. This strategy was used for detection of methyl-paraoxon or chlorpyrifos [267]. The micro-spot structure is mostly used in fluorescence-based biosensors. In these sensors, the fluorescent probes, such as tetraphenylethene (TPE) functionalized with maleimide group [268] or ZnCdSe\CdTe\Zn-nanoporphyrin [269], were applied for the detection of diazinon and dimethoate, dichlorvos, and demeton, respectively. Studies have also been carried out on micro-spot sensors with the surface-enhanced Raman scattering mechanism, modifying the paper substrate with a solution of gold nanoparticles coating by Raman probe (e.g., 4-mercaptobenzoic acid) [270]. The prepared sensor is exposed to a sample containing methyl parathion, having a linear response at the analyte concentration range of 0.018–0.354 μg·cm^−2^, and detection limit of 0.011 μg·cm^2^ [270].

Enzyme biosensors have also been popular in the electrochemical studies of organophosphates [271]. Compared to colorimetric methods, the electrochemical biosensors are resistant to interference from the color or opacity of the sample matrix [271]. The cholinesterase enzymes are used in the structure of these biosensors. For example, butyrylcholinesterase was used for detection of methyl parathion with a potentiometric method [140] or simultaneous determination of various type of pesticide with an amprometric method [272]. In the later, the graphite working electrodes were modified by Prussian blue nanoparticle integrated with carbon black, preventing the precipitation of thiol products on the electrode surface while also increasing the oxidative current relative to unmodified electrodes. Additionally, the current is measured in the presence and absence of the analyte using a pad, reducing the analysis cost and time [272]. Despite their applicability in detecting organophosphates, cholinesterases are also prone to other interfering species in the medium [215]. Consequently, cholinesterases do not show good selectivity against enzymes such as phosphotriesterase (PET), and interact only with compounds containing specific ester bonds that are also found in organophosphates [273]. Hondred et al. have used PET in the fabrication of the paraoxon biosensor, and employed the inkjet maskless lithography method for designing the first printed graphene sensor [274]. By designing an unmodified enzyme immunosorbent assay, one can use a graphene electrode to detect parathion, according to Mehta et al. [275]. In this respect, anti-parathion antibodies were attached to the electrode surface by means of amines-based material (e.g., 2-aminobenzyl amine) as a mediator. The sensor response was recorded by an impedance method, resulting in a detection limit of 0.052 ng·L^−1^ for tomato and carrot samples [275].

**Figure 6:**
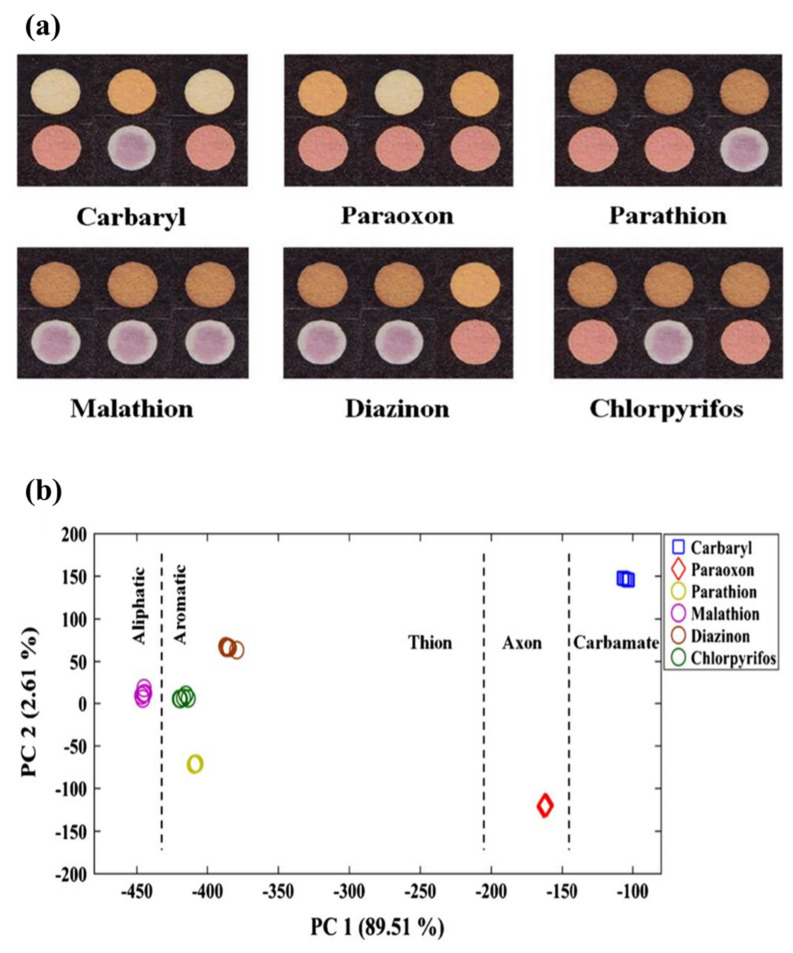
Paper-based electronic tongue for detection of pesticides. This sensor can discriminate carbamate, oxon organophosphate, and thion organophosphate from each other. It can also separate aliphatic thions from non-aliphatic species. This figure illustrates the actual sensor response (**a**) and PCA score plot (**b**) which are shown the discriminatory ability of proposed sensor (Reprinted with permission from [276], copyright (2020) Springer Nature).

Enzyme-based colorimetric and electrochemical methods do not have good selectivity for similar organophosphates, preventing the possibility of their simultaneous determination in a real sample containing several species of organophosphates. In addition, despite the high specificity of immunoassay methods, it is difficult to immobilize multiple antibodies on a piece of paper. In order to simultaneously monitor organophosphates by paper sensors, Bagheri research group has proposed a nanoparticle-based color sensor array [276]. They used modified gold and silver nanoparticles, generating a fingerprint response for all pesticides studied. As seen in Figure 6, the sensor response show a discrimination between pesticides from non-pesticides, carbamates from organophosphate, oxones from thions, and aliphatic thions from non-aliphatic species, providing a good sensitivity in the order of nanogram per milliliter for separate quantitative and simultaneous analysis of pesticides [276]. Elsewhere, Bagheri et al. have used nanoparticle-based origami paper structures to analyze sulfur-containing organophosphates in the vapor phase, simultaneously [277].

**Table 2:** Different type of paper-based biosensor for organophosphate detection.

Type of OP	Device Structure	Bioreceptor	Detection Method	Sensing Element	Media	Linear Range	Detection Limit	Ref.
Parathion	Origami	Enzyme	Potentiometric	butyrylcholine-sensitive membrane		0.1–1.0 nm	0.06 nm	[140]
Parathion	Printed electrode	Anti-parathion	Impedimetric	Gr/ABA	Tomato and carrot	0.1–1000 ng·L^−1^	52 pg·L^−1^	[275]
Parathion	Micro spot	Free	SERS	Au NPs\4-MBA	apple	0.018–0.354 μg·cm^−2^	0.011 μg·cm^−2^	[270]
Paraoxon	Printed electrode	Enzyme	Amperometric	PtNP-IML-PGE/GA	Soil and water	0.1–1.0 nM	3 nM	[274]
Malathion	Printed electrode	Mitochondria	Voltammetric	Quinone			20 nM	[278]
Chlorpyrifos	3D paper	Enzyme	Colorimetric	Indoxyl acetate		0–25.0 ppm	8.60 ppm	[262]
Trichlorfon	Microfluidic assay	Free	Colorimetric	ammonium molybdate method	Pak choi, broccoli, swamp, cabbage		1.65 μg·mL^−1^	[279]
Diazinon	Micro spot	Enzyme	Fluorimetric	TPE	Human serum	0.3–5.0 ng·mL^−1^	0.23 ng·mL^−1^	[268]
Paraoxon Chlorpyrifos	Micro spot	Enzyme	Colorimetric	Nanoceria	Human serum	0–100.0 ng·mL^−1^ 0–60 ng·mL^−1^	18.0 ng·mL^−1^ 5.3 ng·mL^−1^	[267]
Phorate, avermectin, imidacloprid	3D paper	Enzyme	Colorimetric	Indophenol acetate	Lettuce and rice			[280]
Chlorpyrifos parathion methyl-parathion malathion fenitrothion carbaryl	Origami	Enzyme	Colorimetric	indophenol acetate	Cabbage extracts	5.0–100.0 μg·mL^−1^ 1.0–8.0 μg·mL^−1^ 0.5–6.0 μg·mL^−1^ 0.5–6.0 μg·mL^−1^ 0.5–6.0 μg·mL^−1^ 1.0–8.0 μg·mL^−1^	3.3 μg·mL^−1^ 0.52 μg·mL^−1^ 0.46 μg·mL^−1^ 0.45 μg·mL^−1^ 0.47 μg·mL^−1^ 0.51 μg·mL^−1^	[263]
Dimethoate dichlorvos, demeton	Micro spot	Free	Fluorimetric	CdTe QDs/ZnCdSe QDs/Nano-ZnTPyP	Apple and cabbage			[269]
paraoxon 2.4-DCPA atrazine	Origami	Enzyme	Electrochemical	GP/CB/PBNPs GP/CB GP/CB	River water	2–20 ppb 100–600 ppb 10–100 ppb	2 ppb 50 ppb 10 ppb	[272]
Carbaryl, paraoxon, parathion, malathion, diazinon, chlorpyrifos	Origami	Array-based e-tongue	Colorimetric	AuNPs and AgNPs	Tap water, apple juice, rice	35.0–2500.0 ng·mL^−1^ 25.0–5000.0 ng·mL^−1^ 35.0–5000.0 ng·mL^−1^ 20.0–2500.0 ng·mL^−1^ 50.0–7500.0 ng·mL^−1^ 40.0–2500.0 ng·mL^−1^	29.0 ng·mL^−1^ 22.0 ng·mL^−1^ 32.0 ng·mL^−1^ 17.0 ng·mL^−1^ 45.0 ng·mL^−1^ 36.0 ng·mL^−1^	[276]
Parathion, malathion, diazinon, chlorpyrifos	Origami	Array-based e-nose	Colorimetric	AuNPs and AgNPs	Ambient air	70–1000 ng·mL^−1^ 110–810 ng·mL^−1^ 90–730 ng·mL^−1^ 130–730 ng·mL^−1^	58.0 ng·mL^−1^ 103.0 ng·mL^−1^ 81.0 ng·mL^−1^ 117.0 ng·mL^−1^	[277]

OP: Organophosphate, 2-ABA: 2-Aminobenzyl amine, Gr: Graphene, PtNPs: Platinum nanoparticles, IML: Inkjet maskless lithography, PGE: Patterned graphene electrodes, GA: Glutaraldehyde, SERS: Surface-enhanced Raman scattering (SERS), TPE: Tetraphenylethylene, e-tongue: electronic tongue, 2.4-DCPA: 2,4-dichlorophenoxyacetic acid, GP: Graphite, CB: Carbon black, PBNPs: Prussian blue nanoparticle.

### 10.3. Pathogen Bacteria Detection

Pathogenic bacteria is one of the causes of human disease leading to infections in the body or even death [277]. Bacterial infections can originate from food, water, or air after entering the body, infecting areas such as the lungs, stomach, intestines, skin, kidneys, bladder, and even the blood [281]. The bacterial infection in its mildest form leads to intoxication with fever, chills, and fatigue [281]. Annually, two million people are killed by waterborne pathogens alone [282]. Pathogenic bacteria can be classified into two categories: gram-positive and gram-negative [283]. Compared to gram-negative bacteria, the cell wall of gram-positive bacteria has a thick peptidoglycan layer, without the outer membrane. This causes the gram-positive and gram-negative bacteria to turn purple and pink in the gram staining test, respectively [283]. Bacteria such as *Staphylococcus aureus*, *Listeria monocytogenes*, *Streptococcus agalactiae*, and *Enterococcus faecalis* are in the gram-positive group, and bacteria such as *Escherichia coli*, *Klebsiella pneumoniae*, *Proteus mirabilis*, *Enterobacter aerogenes*, and *Pseudomonas aeruginosa* are in the gram-negative group [282].

The most common clinical methods used to detect bacterial infections are as follows: culturing, enzyme-linked immunosorbent assay, and polymerase chain reaction [284]. While the accuracy and sensitivity of these methods are very good and have been used as a gold standard approach in medical centers for many years, they take a long time (at least 48 h) to determine the infection and the type of bacteria [285]. The diagnosis of infectious diseases in a short time is of great importance. For this reason, many efforts have been made to develop rapid test sensors, a considerable part of which are based on paper biosensors. A sensor is highly efficient when it can detect fewer bacteria in the shortest possible time.

Thus far, the use of paper biosensors has been reported in the study of food spoilage (cold cuts, sausages, beef, and pork), beverages (orange juice, milk, and drinking water), vegetables and fruits (cucumber and lettuce), and biological samples (serum and urine), as can be observed in Table 3. Enzymes and aptamers have been mainly used as bioreceptors in the colorimetric detection of bacteria (Table 3). For example, Creran et al. used *β*-galactosidase (*β*-gal) enzyme to determine *Escherichia coli* XL1 and *Bacillus subtilis* in drinking water samples [286]. This enzyme can catalyze the conversion reaction of yellow chlorophenolred-β-D-galactopyranoside (CPRG) to its purple state. In this respect, the anionic enzyme is first electrostatically attached to a gold nanoparticle with a positive electric surface. In the presence of analyte, the nanoparticle tends to interact with bacteria more, resulting in the separation of the enzyme from the complex structure, while also reacting with CPRG to change the sensor color from yellow to purple [286]. In addition to *β*-gal, Jokerst et al. have employed phosphatidylinositol-specific phospholipase C (PI-PLC) and esterase [287]. The PI-PLC catalyzes the reaction of 5-bromo-4-chloro-3-indolyl-myo-inositol phosphate (X-InP) to the indigo form, and the esterase catalyzes the reaction of 5-bromo-6-chloro-3-indolyl caprylate to its purple state. The *β*-gal, PI-PLC, and esterase enzymes have been used to detect *Escherichia coli*, *Listeria monocytogenes*, and *Salmonella enterica*, respectively [287]. According to Jokerst et al., by combining paper biosensors and polymerase chain reaction (PCR), it is possible to detect 10 Cfu/cm^2^ in less than 12 s [287]. The detection mechanism of *Staphylococcus aureus*, with a sensor proposed by Suaifan et al., is based on the proteolytic activity of *S. aureus* proteases on a magnetic nanobeads-peptide probe being immobilized on the gold platform via an Au-S connection [288]. During the reaction, magnetic nanobeads detached from the surface are separated from the gold platform using an external magnet, resulting in the appearance of yellow color. The time required for this measurement has been reported to be 1 min [288]. The α-glucosidase enzyme is secreted by various species of *Cronobacter* spp., catalyzing the 5-bromo-4-chloro-3-indolyl-α-D-glucopyranoside (XαGlc) color species to the form of indigo, which can be used in a paper sensor. This can allow for the detection of the presence and amount of different species of *Cronobacter* spp., up to a concentration of 10 Cfu/cm^2^ in 10 h [289].

**Figure 7:**
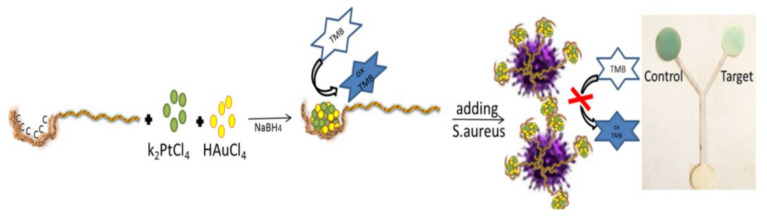
The nanozyme-based sensor for detection of *Staphylococcus aureus*. Au/Pt bimetallic nanoclusters synthesized by cytosine-rich, single-strand DNA show enzyme-like activity, to catalyze TMB to its oxidized form in the presence of H_2_O_2_; the color of sensor changes from colorless to dark blue. In the presence of bacteria strain, the nanoclusters interact with the bacteria, and the redox reaction of TMB is inhibited, leading to a reduction in the intensity of the blue color (reprinted with permission from [290], copyright (2020) Elsevier).

The detection of *Staphylococcus aureus* has been investigated using the nanozyme nature of nanoclusters. In one study, Bagheri Pebdeni et al. synthesized Au/Pt bimetallic nanoclusters using cytosine-rich, single-strand DNA, catalyzing TMB in its oxidized form in the presence of H_2_O_2_, due to the peroxidizing properties [290]. This causes the sensor to change from colorless to dark blue. The catalytic activity of the nanoclusters changes in the presence of bacteria, leading to the production of less oxidized TMB and, consequently, reducing the intensity of the blue color [290]. The schematic diagram for the working mechanism of this sensor is represented in Figure 7.

Unlike colorimetric methods, electrochemical techniques mostly employ antibodies and aptamers as bioreceptors (Table 3). In most cases, the detection element is a graphene electrode (or its derivatives) coated on paper by screen printing. To increase sensor performance, the graphene electrode can be modified by some compounds, such as gold nanoparticles and polymers. In this direction, using an electrodeposition technique, Wang et al. coated gold nanoparticles on an electrode surface made of reduced graphene oxide (rGO). After immobilizing the antibody on the electrode surface using the biotin-streptavidin system, they used *Escherichia coli O157: H7*, which was detected by an amperometric biosensor with a detection limit of 1.5 × 10^2^ Cfu/mL [291]. Neutral red (NR)-modified graphene electrodes have been used in the design of sandwich immunoassay systems, according to Mo et al. [292]. In this respect, polyaniline (PANI) was precipitated on a carbon electrode, and the primary antibody (Ab1) was bound to the polymer via gold nanoparticles. On the other hand, rGO-NR was electrostatically bound to Au@Pt nanoparticles with a negative surface charge. The secondary antibody (Ab2) was bound to Au@Pt nanoparticles. Bacteria were sandwiched between these two layers, thereby increasing the sensitivity of the electrochemical signal [292].

Graphene electrodes can be functionalized with thermoresponsive polymers, such as poly (N-isopropylacrylamide) (PNIPAm). These polymers are sensitive to the physiological temperature (37 °C). Khan et al. have used the PNIPAm polymer to fabricate the PNIPAm-graphene nanoplatelet electrode nanocomposite, being precipitated on a gold substrate coated on paper [293]. The fibrous structure of the nanocomposite is such that it receives bacterial cells without the need for the presence of antibodies, thus changing the resistance of the gold platform. The resulting nanocomposite sensor was used to detect *S. mutans*, *B. subtilis*, and *E. coli* belonging to gram-positive and gram-negative bacteria groups, respectively. Moreover, the detection time for this sensor was reduced to 10 min [293].

The electrochemical behavior of graphene electrodes, modified by a specific bioreceptor, can be different from each other in the detection of bacteria. For example, Hernández et al. immobilized an aptamer on GO and rGO [294]. A covalent approach was used to immobilize the bioreceptor on GO, whereby amide bonds formed between the carboxyl group of GO and the amino groups of the aptamer. Alternatively, the rGO is non-covalently modified by the pyrenyl aptamer head. The aptamer has a strong tendency to interact with bacteria, dominating π–π interactions to separate its negatively ionized phosphodiester groups from the electrode surface in the presence of analyte. In turn, this changes the electrode potential. The experimental observations have shown that the modified rGO electrode has less noise and a higher detection limit than the GO. Hernández et al. have used this potentiometric system to detect *Staphylococcus aureus*, with a detection limit of 1 Cfu/mL in less than 2 min [294].

Sometimes antibodies can be covalently attached to carbon nanotubes with the help of a mediator, in order to detect bacteria. The covalent bonding reduces the time of the functionalization process of the paper surface and increases the stability of the sensor. In a study by Bhardwaj et al. [295], bacterial antibodies were immobilized on single-walled carbon nanotubes, using N-(3-dimethylaminopropyl)-N′-ethylcarbodiimide hydrochloride/N-hydroxysuccinimide mediator, allowing for the detection of *S. aureus* in milk samples. Accordingly, bacteria could be detected in the linear range of 10−10^7^ Cfu/mL in less than 30 min. The resulting sensor can determine *S. aureus* in the presence of other bacterial species, including *Escherichia coli B*, *Bacillus subtilis*, and *S. epidermidis*, providing excellent selectivity, due to the use of the specific antibody [295].

**Figure 8:**
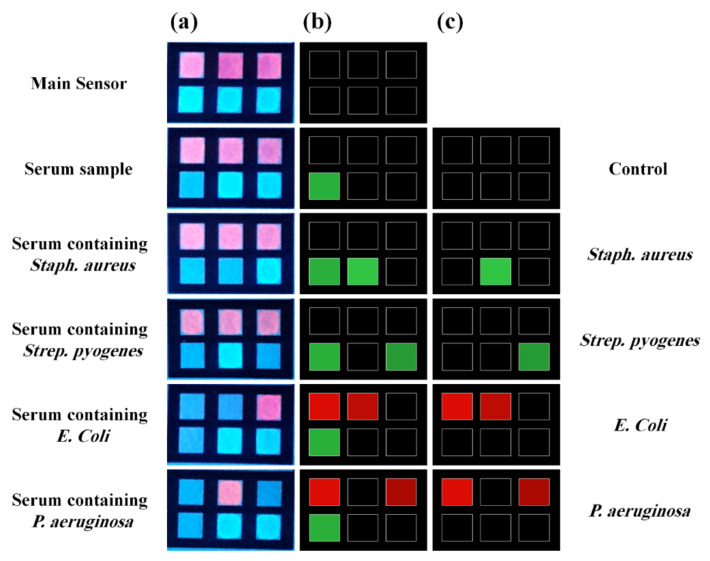
A fluorimetric, paper-based sensor for detecting of bacteria-caused sepsis. Array was constructed by gold and copper nanoclusters, which are synthesized by proteins. The interaction between detection elements and serum contaminated with bacteria leads to turning off fluorescence emission. This figure represents the actual response of sensor (**a**) and respective difference maps (**b**) for the control and each bacteria strain. (**c**) The response of sensor after eliminating the effect of the pure serum sample (reprinted with permission from [307], copyright (2021) Elsevier).

In recent years, with the development of sensor arrays, it has become possible to detect and determine bacteria simultaneously [296,297,298,299,300]. The detection mechanism is based on changes in the optical or electrical properties of the indicator after the interaction with bacteria or metabolites emitted from the bacterial growth. The detection elements can be organic semiconductors, conductive polymers, polymer carbon black composites, and dyes [301,302,303,304,305,306]. In a study by Bordbar et al. [282], the sensor array was composed of sixteen nanoparticles (eight gold and eight silver nanoparticles), each of which was synthesized by 8 coating agents, consisting of protein, polymer, surfactant, carbohydrate, and amino acid exposed to ten strains of bacterial. The detection process was evaluated in ambient water samples and 300 urine samples of ill and healthy persons. It was reported that the sensor array was capable of determining 10^2^ Cfu/mL during 10 min in the culture media, 50 min in the aqueous sample, and 30 min in the urine sample. This sensor can be used to detect urinary tract infections with high accuracy [282]. Moreover, it is possible to use a paper sensor array in the diagnosis of sepsis in children. This has been designed by Sheini, through immobilizing gold and silver metal nanoclusters on Whatman paper [307]. The resulting sensor scan determine sepsis-causing bacteria, including *Staphylococcus aureus*, *Streptococcus pyogenes*, *Escherichia coli*, and *Pseudomonas aeruginosa*, in pediatric serum samples and provide a detection limit of less than 100 Cfu/mL. In this case, changes in fluorescence emission intensity were recorded in less than 15 s, with the help of a hand-held UV lamp and a smartphone [307]. One can see the results of this study in Figure 8.

**Table 3:** Different type of paper-based biosensor for bacteria detection.

Type of Bacteria	Device Structure	Bioreceptor	Detection Method	Sensing Element	Media	Detection Limit	Time	Ref.
*E. coli* O157:H7	paper electrode	Antibody	Impedimetric	rGOPE/AuNPs	Ground beef and cucumber	1.5 × 10^−2^ Cfu mL^−1^		[291]
*E. coli* XL1	Inkjet-Printed test strip	Enzyme	Colorimetric	CPRG	Drinking water	10^2^ bacteria mL^−1^	5 min	[286]
*E. coli*	Micro spot	Aptamer	Fluorimetric	Fluorogenic DNAzyme probe	Milk, apple juice, and drinking water	100 cells mL^−1^		[308]
*E. coli* K12	Origami	Aptamer	Colorimetric	TMB, Hemin, H_2_O_2_	Juice and milk	10^3^ Cfu mL^−1^	35 min	[309]
*E. coli* O157:H7	Printed electrode	Antibody	Electrochemical	SPCE-PANI-AuNPs-Ab1 and PANI-rGO-NR-Au@Pt-Ab2	Milk and pork	2.84 × 10^3^ Cfu mL^−1^	60 min	[292]
*S. aureus*	Paper electrode	Aptamer	Potentiometric	GO or rGO		1 Cfu mL^−1^	1–2 min	[294]
*S. aureus*	Test strip	Enzyme	Colorimetric	Magnetic nanobeads/Peptide	Ground beef, turkey sausage, lettuce, milk, and dust samples	7 Cfu mL^−1^ (Pure broth culture) 40 Cfu mL^−1^ (food products) 100 Cfu mL^−1^ (environmental samples)	1 min	[288]
*S. aureus*	Paper electrode	Antibody	Electrochemical	SWCNT	Milk	13 Cfu mL^−1^	30 min	[295]
*S. aureus*	Microfluidic assay	Nanozym	Colorimetric	DNA-Au/Pt BMNCs, H_2_O_2_, and TMB	Milk, orange juice, and human serum	80 Cfu mL^−1^	60 min	[290]
*E. faecalis*	Paper strip	Free	Colorimetric	Resazurin			10 min	[310]
*Salmonella typh*.	Paper strip	Antibody	Potentiometric	PAMAM(NH_2_)_64_/GA/Ab	Apple juice	5 cells mL^−1^	<1 h	[311]
Each type	Printed electrode	Protein	Impedimetric	Con A	Water	1.9 × 10^3^ Cfu mL^−1^		[312]
*Cronobacter* spp.	Micro spot	Enzyme	Colorimetric	XαGlc		10 Cfu cm^−2^	10 h	[289]
*E. coli* O157:H7, *Salmonella*, and *Listeria*	Micro spot	Enzyme	Colorimetric	CPRG, Magenta caprylate, and X-InP	Bologna	10 Cfu cm^−2^	8 h	[287]
*E. coli*, *S. mutans*, and *B. subtilis*	Paper electrode	Polymer	Thermoelectrochemical	Gr-PNIPAm-Au	Autoclave, tap and lake waters, and milk	5 cells mL^−1^	less than 10 min	[293]
*S. aureus*, *Listeria*, *E. coli*, proteus, *klebsiella*, *E. aerogenes*, *P. aeruginosa*, *E. faecalis*, *S. agalactiae*, MRSA	Micro spot	Array-based E.nose	Colorimetric	AuNPs and AgNPs	Tap and mineral water, and human urine	1.0 × 10^2^ Cfu mL^−1^	50 min (water) 30 min (urine)	[282]
*S. aureus* *S. pyogenes* *E. coli* *P. aeruginosa*	Microfluidic assay	Array-based E.tongue	Fluorimetric	Protein based Au and CuNCs	Serum (for detecting sepsis)	43.0 Cfu mL^−1^ 63.5 Cfu mL^−1^ 26.0 Cfu mL^−1^ 47.0 Cfu mL^−1^	15s	[307]

*E. coli*: *Escherichia coli*; rGOPE: reduced graphene oxide paper electrode; CPRG: chlorophenol red-β-D-galactopyranoside; NR: neutral red; PANI: polyaniline; SPCE: screen-printed carbon electrode; Ab: antibody; *S. aureus*: *Staphylococcus aureus*; GO: graphene oxide; rGO: reduced graphene oxide; SWCNT: single walled carbon nanotube; BMNCs: bimetallic nanoclusters; TMB: 3,3′,5,5′-tetramethylbenzidine; *E. faecalis*: *Enterococcus faecalis*; *Salmonella typh*.: *Salmonella typhimurium*; GA: glutaraldehyde; PAMAM: poly(amidoa-mine); Con A: lectin concanavalin A; XαGlc: 5-bromo-4-chloro-3-indolyl-α-D-glucopyranoside; *Listeria*: *listeria monocytogenes*; X-InP: 5-bromo-4-chloro-3-indolyl-myo-inositol phosphate; Magenta caprylate: 5-bromo-6-chloro-3-indolyl caprylate; PNIPAm: poly(N-isopropylacrylamide); *B. subtilis*: *Bacillus subtilis*; *S. Mutans*: *Streptococcus mutans*; MRSA: methicillin-resistant Staphylococcus aureus; *S. agalactiae*: *Streptococcus agalactiae*; *klebsiella*: *klebsiella pneumonia*; *E. aerogenes*: *Enterobacter aerogenes*; *P. aeruginosa*: *Pseudomonas aeruginosa*.

### 10.4. Heavy Metal Ions Detection

The waste from mines and other industrial centers, fertilizers, and pesticides, as well as fuels and pollutants from vehicles, ships, and heavy machinery, can be sources of heavy metal ions [313]. The entry of the metal ions into the environment reduces the quality of water, air, and soil, which in turn leads to the extinction of various plant and animal species [314]. When the human body is exposed to the heavy metal ions, they disrupt the body’s immune system, resulting in respiratory, skin, digestive, kidney, and liver problems and even cancer, in acute cases [315]. As mentioned in Table 4, most studies on heavy metal poisoning have been performed for drinking and ambient water samples. According to the protocol of the World Health Organization, the permissible limit of heavy metals in aqueous samples should be in the range of 0.01−0.05 mgL^−1^ [315].

Mercury (II) ions (Hg(II)) is a heavy metal ion with a limit of 6 µgL^−1^ in aqueous samples [316]. To determine Hg(II) ions by paper sensors, the following three structures have been used: micro-spot, microfluidic, and distance quantitation (Table 4). By preparing a paper aptasensor-based micro spot, Chen et al. determined Hg(II) ions with a detection limit of 50 nm in river and pond water samples [317]. A single-stranded DNA is physically bound to Au nanoparticles. In the presence of the analyte, the structure of the single-stranded DNA changes to the hairpin structure, due to the binding of thymine–Hg(II)–thymine, giving rise to the separation of the aptamer from the nanoparticles. By adding salt to the reaction medium, the electron repulsion of the bare nanoparticles is reduced, causing the nanoparticles aggregation and leading to a change in the color of the sensor from red to blue [317]. Pt nanoparticles can have peroxidase-like properties, catalyzing the TMB oxidation reaction to its oxidized form and changing the color of the sensor from colorless to blue [318]. Furthermore, Pt nanoparticle can be compounded with the Hg(II) ion, producing Hg-Pt alloy [318]. As a result, the presence of Hg(II) in the reaction medium inhibits the enzymatic activity and reduces the intensity of the blue color of the sensor [318]. Chen et al. used this mechanism to determine Hg(II) ions in drinking and pond water samples, having a detection limit of 0.01 µM, performed on the micro-spot paper [318]. Some mercury sensors have been fabricated based on the length measurement. The sensing element in these sensors can be a colorimetric reagent (e.g., dithizone) [319] or a fluorescent compound (e.g., nitrogen-doped carbon dots (NCDs)) [316]. In the former, the color change (from yellow to purple) is due to the formation of dithizone-Hg precipitate on the surface of the paper, detecting Hg(II) ions with a detection limit of 0.93 μgmL^−1^ [319]. In the latter, the analyte causes the fluorescence of NCDs to be quenched. This fluorescence quench is due to the formation of a non-fluorescent Hg-NCDs complex, arising from the covalent interaction of NCDs electrons with empty orbitals of the Hg(II) ion. The electrons of NCDs are supplied by the C=N, C=O, and C−OH functional groups in the carbon dot structure. On the other hand, the surface of CDs has been modified by ethylenediamine, so that the mercury tends to interact with the nitrogen existing in this compound [316]. Based on the observations by Ninwong et al., the detection limit of the latter method is 0.005 μgmL^−1^, being considerably smaller than that of the colorimetric method [316]. Nashukha et al. have designed a membraneless gas separation µPAD for determining Hg (II) ions [320]. The proposed sensor consists of donor and acceptor layers together with an interlayer space. In the donor layer, analyte with iodide added in large quantities to the medium forms a water-soluble HgI_4_^2−^ complex. The remaining iodide reacts with the iodate (already immobilized on the paper), resulting in the production of volatile iodine. In turn, the iodine vapors pass through the interlayer space and penetrate the acceptor layer, so that they can react with the iodide-starch indicator to form a tri-iodide starch complex. Accordingly, the color of the acceptor layer changes to purple, corresponding to the concentration of Hg(II) ions [320].

Based on the US Environmental Protection Agency (EPA), the permissible level of copper (II) (Cu(II)) ions in a drinking water sample is 1.3 ppm [321]. Numerous paper sensors have been designed to detect Cu(II) ions, based on colorimetric, fluorometric, and electrochemical methods. In the colorimetric method, the paper sensor can have 2D microfluidic, lateral flow, or 3D origami structure (Table 4).

Ratnarathorn et al. have used a 2D microfluidic structure to measure Cu(II) ions in tap and pond water samples [322]. The measurement was performed based on the accumulation of silver nanoparticles, whose surface was simultaneously modified by homocysteine and dithiothreitol. The detection limit of this method was reported to be 7.8 nM [322]. Quinn et al. introduced distance-based µPADs, whose detection zone was coated with dithiooxamide [321]. In this regard, the metal ion is separated from the sample texture using the solid-phase extraction method, eliminating the interfering effect of the texture, while also causing the metal ion to be pre-concentrated. This improves the detection limit of the method up to 20 ppb [321]. One of the disadvantages of microfluidic methods is that the sensing element in the sensor moves around the detection zone with the sample stream, making the monitoring of the sensor response erroneous [276]. Moreover, a part of the sensor response disappears in the microfluidic methods. Polyvinylchloride can be used to stabilize the color on the paper uniformly [62]. Sharifi et al. used this mechanism to quantitatively determine Cu(II) ions with pyrocatechol violet and chrome azurol S indicators [62]. In the origami structure, a waste layer is designed to collect the ions unreacted with the color indicator, improving the detection limit of Cu(II) ion measurements with organic color indicators to less than 2 mgL^−1^ [62]. To electrochemically study Cu(II) ions, Wang et al. designed a three-electrode paper device, using a magnetron sputtering technology (MST) on a nitrocellulose paper substrate [323]. The MST creates a uniform porous structure, without the need for additional surface modification. The measurement was performed by square-wave stripping voltammetry, with a detection limit of 2 μgL^−1^ [323].

The fluorescence detection was performed with sensing elements, such as quantum dots and nanoclusters. At Fang’s suggestion, a distance-based µPAD was designed with the help of BSA-Au nanocrystals [324]. The fluorescence of the nanocluster is quenched in the presence of metal ions, allowing for adjusting the detection limit by controlling the water absorbed on the pad. Therefore, it can easily be used to detect Cu(II) ions in complex samples such as blood, soil, and sewage. The sensor response is not affected by a mixture of other ions, providing sensitivities in the range of 5 μM [324].

The poisoning by cadmium (II) (Cd(II)) ions can cause serious damage to the liver and kidneys [325]. The permissible limit of Cd(II) in the aqueous sample has been reported to be between 3 and 5 ppb [326]. It is possible to specifically determine Cd(II) ions by a lateral flow immunoassay method. López Marzo et al. [327] proposed a method based on a competitive reaction between Cd−EDTA complex and Cd−EDTA−BSA−AuNP to interact with the same active site of antibody immobilized on the test line. In the absence of cadmium (II) ion, a distinct red color is detected in the test line whose intensity decreases with increasing thee cadmium concentration and replacement of Cd−EDTA with conjugated nanoparticles [327]. In another study, the lock-and-key theory has been employed to design the sensor. The receptor was an ion-imprinted polymer that reacted specifically with Cd(II) ions. Increasing the concentration of metal ions changed the color of the sensor with an origami paper structure from yellow to red. The detection limit of the measurement was 0.4 ng·mL^−1^ [328].

Since the permissible level of lead (II) (Pb(II)) ions in ambient samples is 15 ppb [329], POCT has been designed to detect them in a variety of paper samples. In this design, carbon dots can act as sensing elements. Gupta et al. have synthesized CDs by heating a biological medium such as potato-dextrose agar (PDA) in the microwave [330]. The chemical structure of these CDs comprises hydroxyl and carboxylic groups, forming a stable complex with Pb(II) ions which results in fluorescence quenching of CDs. The detection limit of this micro-spot paper sensor was 110 pM [330]. Wang et al. employed a combination of two CDs with blue and red fluorescence emission as the sensing element [331]. The blue CDs were synthesized by a combination of sodium citrate and polyacrylamide, having amine and carboxylic groups. The red CDs contained amine groups related to p-phenylenediamine (p-PDA). In the presence of Pb (II) ions, the fluorescence of blue CDs is quenched due to the interaction of lead with the carboxylic groups, whereas the emission intensity of red CDs increases, providing a detection limit of 2.8 nM [331].

Compared to other metal ions, the presence of chromium (III) (Cr(III)) ions is recommended in the human diet at concentrations of 50–200 mgdL^−1^, influencing the glucose, lipid, and protein metabolism efficiently [332] However, at high concentrations, Cr(III) ion can damage the cell by binding to DNA [332]. In order to determine Cr(III) ions, Elavarasi et al. proposed a paper sensor based on gold nanoparticles, synthesized by citrate, without the coating of any other chemical compound on their surface [333]. In the presence of metal ions, gold nanoparticles accumulate and change color from red to blue. This accumulation is due to the tendency of chromium (III) ions to interact with citrate oxygen groups, demonstrating a high selectivity to the ions [333].

The paper sensors are capable of detecting multiple metal ions in ambient samples simultaneously. To this end, Feng et al. [334] have designed a sensor array consisting of nine indicators from the derivatives of 4,4-difluoro-4-bora-3a,4a-diaza-s-indacen, allowing for qualitatively and quantitatively determining seven metal ions, including cobalt(II), mercury(II), copper(II), cadmium(II), nickel(II), zinc(II), and silver(I) in aqueous waste samples with a detection limit of 10^−7^ M. The designed sensor comprised an absorber layer located below the detection area, absorbing 800 μL of the solution containing metal ions to be in contact with fluorescence indicators [334]. In another study, Feng et al. have used a microfluidic structure to simultaneously detect metal ions. The detection process was performed by changing the color of pyridylazo derivatives. As an advantage, this detection method does not require tools to inject the sample onto the paper surface as it is in constant contact with the indicator. In the aforementioned studies, cross reactive indicators were used to design the sensor [335]. Nevertheless, in some cases, it is possible to use indicators that specifically interact with a metal species. For example, nickel(II), chromium(VI), mercury(II), and iron(II) ions can be detected using Dimethylglyoxim [336,337,338], 1,5-diphenylcarbazide [336], Michler’s thioketone [336], bathophenanthroline [337], or 10-phenanthroline [338], respectively. To detect copper (II) ions, the following materials can be used: diethyldithiocarbamate [337], bathocuproine [338], and cuprizone [339]. Sensors with strip or micro-spot structure based on these indicators are employed to evaluate the contamination of drinking and environmental water samples [340,341]. Most of the sensor designs use image analysis software with the capability of being installed on a smartphone, thus analyzing the corresponding color changes.

**Table 4:** Different type of paper-based sensor for heavy metal detection.

Type of Metal Ions	Device Structure	Detection Method	Sensing Element	Media	Linear Range	Detection Limit	Ref.
Hg(II)	Microfluidic assay	Colorimetric	KI, KIO_3_, Starch	Soil	50–350 mg L^−1^	20 mg L^−1^	[320]
Hg(II)	Distance-based sensor	Fluorimetric	NCD	Drinking, pond, and tap waters	0.5–25 mg L^−1^	0.5 mg L^−1^	[316]
Hg(II)	Distance-based sensor	Colorimetric	Dithizone	whitening cream	1–30 µg mL^−1^	0.93 µg mL^−1^	[319]
Hg(II)	Micro spot	Colorimetric	PtNPs-TMB	Pond and tap waters	0.025–0.5 μM	0.01 μM	[318]
Hg(II)	Micro spot	Colorimetric	ssDNA-AuNPs	Water	0–2 µM	50 nM	[317]
Cu(II)	Microfluidic assay	Colorimetric	Hcy-DTT-AgNP	Water	7.8–62.8 μM	7.8 nM	[322]
Cu(II)	Microfluidic assay	Fluorometric	CdTe QDs-Cu-IIP	Sea and lake waters	0.032‒3.2 mg L^‒1^	0.012 mg L^‒1^	[342]
Cu(II)	Distance-based sensor	Fluorometric	BSA-AuNCs		5–500 µM	5 µM	[324]
Cu(II)	Distance-based sensor	Colorimetric	Dithiooxamide	Drinking water	20–500,000 ppb	20 ppb	[321]
Cu(II)	Paper electrode	SWSV		Lake waters	5–1000 µg L^−1^	2 µg·L^−1^	[323]
Cu(II)	Micro paper	Fluorometric	CdTe QDs/GCNNs	Tea soup, orange juice, and red wine	0.01~5.0 μg·mL^−1^	0.47 ng·mL^−1^	[343]
Cu(II)	Origami	Colorimetric	Chrome azurol S, Pyrocatechol violet	Rain and Tab waters	5.0–1400.0 mg L^−1^ 5.0–200.0 mg L^−1^	1.7 mg L^−1^ 1.9 mg L^−1^	[62]
Cr(III)	Paper strip	Colorimetric	Citrate-AuNPs		10^−3^–10^−6^ M	1.06×10^−7^ M	[333]
Cd(II)	Lateral flow	Colorimetric	Antibody/modified AuNPs	Drinking and tap waters	0.4–10 ppb	0.1 ppb	[327]
Cd(II)	Origami	Colorimetric	Ion imprinted polymer	Water	1–100 ng mL^–1^	0.4 ng mL^–1^	[328]
Pb(II)	Paper strip	Fluorometric	CDs (potato-dextrose agar)	Human cells	Up to 1 μM	106 pM	[330]
Pb(II)	Paper strip	Colorimetric	Mixture of blue CDs and red CDs	Tap water and lake water	15−80 nM	2.89 nM	[331]
Hg(II) Cu(II)	Origami	Fluorometric	CdTe QDs-IIP	Lake and sea waters	0.26–34.0 µgL^−1^ 0.11–58.0 µgL^−1^	0.056 µg·L^−1^ 0.035 µg·L^−1^	[340]
Hg(II) Pb(II)	Origami	ECL	Si@CNCs and Ru@AuNPs	Lake water and human serum	5.0 × 10^−10^ to 1.0 × 10^−6^ M 3.0 × 10^−11^–3.0 × 10^−6^ M	0.2 nM 10 pM	[344]
Cd(II) Pb(II)	Printed electrode	SWSV		Salty soda and dirty ground waters	10–100 ppb 10–100 ppb	2.3 ppb 2.0 ppb	[345]
Ni(II), Cr(VI), Hg(II)	Microfluidic assay	Colorimetric	DMG DPC MT	Lake water		0.24 ppm 0.18 ppm 0.19 ppm	[336]
Fe(II) Ni(II) Cu(II)	Barrier-free patterned paper	Colorimetric	BP DMG DDC	Pond water	0.5–20 ppm 0.4–20 ppm 0.5–20 ppm	0.25 ppm 0.4 ppm 0.5 ppm	[337]
Fe(II) Cu(II) Ni(II)	Micro spot	Colorimetric	BC Phen DMG	Tap and lake water and papermaking wastewater	0.5–500 mg L^−1^ 0.5–500 mg L^−1^ 2–500 mg L^−1^	0.5 mg L^−1^ 0.5 mg L^−1^ 2 mg L^−1^	[338]
Zn(II) Cr(II) Cu(II) Pb(II) Mn(II)	Filter paper	Colorimetric	ZI, cyanide and cyclohexanone DPC CPZ ALS PAN and cyanide	Wastewater	2.00–6.00 mg L^−1^ 0.10–0.50 mg L^−1^ 0.30–8.00 mg L^−1^ 0.08–0.60 mg L^−1^ 0.20–1.00 mg L^−1^	0.63 mg L^−1^ 0.07 mg L^−1^ 0.17 mg L^−1^ 0.03 mg L^−1^ 0.11 mg L^−1^	[339]
Hg(II) Ag(I) Cu(II) Cd(II) Pb(II), Cr(VI) Ni(II)	Microfluidic assay	Colorimetric	CPRG	Distilled, tap, lake, and fall water		0.001 ppm 0.002 ppm 0.020 ppm 0.020 ppm 0.140 ppm 0.150 ppm 0.230 ppm	[341]
Hg(II), Cd(II), Co(II), Cu(II), Ni(II), Zn(II), and Ag(I)	Array-based e-tongue	Fluorometric	DPA derivatives	Wastewater		10^−7^ M	[334]
Hg(II), Cd(II), Co(II), Cu(II), Ni(II), Zn(II), and Pb(II)	Array-based e-tongue	Colorimetric	Pyridylazo compounds	Sewage water		50 µM	[335]

NCD: Nitrogen-doped carbon dots; TMB: 3,3′,5,5′-Tetramethylbenzidine; Hcy: homocysteine; DTT: Dithiothreitol; Cu-IIP: Cu(II) imprinted polymers; BSA: bovine serum albumi; SWSV: square-wave stripping voltammetry (SWSV); GCNNs: graphite carbon nitride; CDs: carbon dots; IIP: ion imprinted polymer; ECL: electrochemiluminescence; Ru@AuNPs: Ru(bpy)_3_^2+^-gold nanoparticles (AuNPs); Si@CNCs: carbon nanocrystals (CNCs) capped silica nanoparticles; ZI: zincon; DPC: 1,5-Diphenylcarbazide; CPZ: cuprizone; ALS: alizarin red S; PAN: 1-(2-pyridylazo)-2-naphthol; MT: Michler’s thioketone; DMG: dimethylglyoxim; BP: Bathophenanthroline; DDC: diethyldithiocarbamate; BC: bathocuproine; Phen: 1,10-phenanthroline; CPRG: chlorophenol red β-galactopyrano-side; DPA: Di-2-picolyamine.

## 11. Conclusions

Due to their simplicity, low cost, easy fabrication, ease of use, and reliable performance, PPOCTs have been welcomed by a large number of research groups. They consume extremely small volumes of analytes, indicators, and receptors. PPOCTs are diverse in design structure and device dimension, having a high ability to detect the analyte in different physical states. However, the performance of these sensors is limited by some disadvantages, such as sample evaporation on the surface of paper, sample entrapment between the paper fibers, low mechanical stability, the infiltration of samples or reagent into the paper layers, appearing coffee effect in the detection zones, variations in the flow rate of samples due to their different viscosity, using readers with low sensitivity, interference of the environmental factors in sensor stability and the optical factors in collection of sensor responses. Limitations can be reduced by some effective solutions, such as sealing paper substrates, modifying the surface of channels, and detecting zones with polymers or proteins to block the paper pores or hydrophobicize the reagents. This can be achieved by using a sealed box to eliminate ambient light (resulting in a reproducible response) and connecting the readers to the wireless system, enabling us to receive the information without the presence in the infected area or to transfer the information to a strategic system. Most of these sensors are commercially available and are used by medical, food, pharmaceutical, and forensics centers, as well as by the environment and the general public. Overall, a variety of paper biosensors have been reported to detect the hazardous contaminations mentioned in this review. However, the development of paper tools for the simultaneous detection of aflatoxins, organophosphates, bacteria, and metal ions (using bioreceptors and paper sensor arrays) could attract the further attention of researchers.

## Data Availability

Not applicable.

## References

[1] Vallero D.A. (2010). Fundamentals of environmental chemistry. Environmental Contaminants: Assessment and Control.

[2] Kudr J., Zitka O., Klimanek M., Vrba R., Adam V. (2017). Microfluidic electrochemical devices for pollution analysis–A review. Sens. Actuators B Chem..

[3] WHO (2019). 7 Million Premature Deaths Annually Linked to Air Pollution.

[4] Landrigan P.J., Fuller R. (2015). Global health and environmental pollution. Int. J. Public Health.

[5] (2013). Lead poisoning and health. Saudi Med. J..

[6] WHO (2016). Arsenic.

[7] Sidhu G.K., Singh S., Kumar V., Dhanjal D.S., Datta S., Singh J. (2019). Toxicity, monitoring and biodegradation of organophosphate pesticides: A review. Crit. Rev. Environ. Sci. Technol..

[8] Bordbar M.M., Hemmateenejad B., Tashkhourian J., Nami-Ana S.F. (2018). An optoelectronic tongue based on an array of gold and silver nanoparticles for analysis of natural, synthetic and biological antioxidants. Microchim. Acta.

[9] George M. (2006). The origins and the future of microfluidics. Nature.

[10] Ren K., Zhou J., Wu H. (2013). Materials for microfluidic chip fabrication. Acc. Chem. Res..

[11] Olanrewaju A., Beaugrand M., Yafia M., Juncker D. (2018). Capillary microfluidics in microchannels: From microfluidic networks to capillaric circuits. Lab Chip.

[12] Hansen S., Wahed A.A. (2020). El Point-of-care or point-of-need diagnostic tests: Time to change outbreak investigation and pathogen detection. Trop. Med. Infect. Dis..

[13] Niculescu A.G., Chircov C., Bîrcă A.C., Grumezescu A.M. (2021). Fabrication and applications of microfluidic devices: A review. Int. J. Mol. Sci..

[14] Vashist S.K. (2017). Point-of-care diagnostics: Recent advances and trends. Biosensors.

[15] Kumar S., Nehra M., Khurana S., Dilbaghi N., Kumar V., Kaushik A., Kim K.H. (2021). Aspects of point-of-care diagnostics for personalized health wellness. Int. J. Nanomed..

[16] Mohankumar P., Ajayan J., Mohanraj T., Yasodharan R. (2021). Recent developments in biosensors for healthcare and biomedical applications: A review. Meas. J. Int. Meas. Confed..

[17] Chen C., Wang J. (2020). Optical biosensors: An exhaustive and comprehensive review. Analyst.

[18] Chen S., Shamsi M.H. (2017). Biosensors-on-chip: A topical review. J. Micromech. Microeng..

[19] Azmi A., Azman A.A., Ibrahim S., Yunus M.A.M. (2017). Techniques in advancing the capabilities of various nitrate detection methods: A review. Int. J. Smart Sens. Intell. Syst..

[20] Nery E.W., Kubota L.T. (2013). Sensing approaches on paper-based devices: A review. Anal. Bioanal. Chem..

[21] Nguyen T., Chidambara V.A., Andreasen S.Z., Golabi M., Huynh V.N., Linh Q.T., Bang D.D., Wolff A. (2020). Point-of-care devices for pathogen detections: The three most important factors to realise towards commercialization. TrAC Trends Anal. Chem..

[22] Cate D.M., Adkins J.A., Mettakoonpitak J., Henry C.S. (2015). Recent developments in paper-based microfluidic devices. Anal. Chem..

[23] Gong M.M., Sinton D. (2017). Turning the Page: Advancing Paper-Based Microfluidics for Broad Diagnostic Application. Chem. Rev..

[24] Bracher P.J., Gupta M., Whitesides G.M. (2009). Shaped films of ionotropic hydrogels fabricated using templates of patterned paper. Adv. Mater..

[25] Tseng S.C., Yu C.C., Wan D., Chen H.L., Wang L.A., Wu M.C., Su W.F., Han H.C., Chen L.C. (2012). Eco-friendly plasmonic sensors: Using the photothermal effect to prepare metal nanoparticle-containing test papers for highly sensitive colorimetric detection. Anal. Chem..

[26] Ngo Y.H., Li D., Simon G.P., Garnier G. (2012). Gold nanoparticle-paper as a three-dimensional surface enhanced raman scattering substrate. Langmuir.

[27] Jarujamrus P., Tian J., Li X., Siripinyanond A., Shiowatana J., Shen W. (2012). Mechanisms of red blood cells agglutination in antibody-treated paper. Analyst.

[28] Bordbar M.M., Tashkhourian J., Hemmateenejad B. (2019). Structural Elucidation and Ultrasensitive Analyses of Volatile Organic Compounds by Paper-Based Nano-Optoelectronic Noses. ACS Sens..

[29] Singh A.T., Lantigua D., Meka A., Taing S., Pandher M., Camci-Unal G. (2018). Paper-based sensors: Emerging themes and applications. Sensors.

[30] Kuswandi B., Ensafi A.A. (2020). Perspective—Paper-Based Biosensors: Trending Topic in Clinical Diagnostics Developments and Commercialization. J. Electrochem. Soc..

[31] Ratajczak K., Stobiecka M. (2020). High-performance modified cellulose paper-based biosensors for medical diagnostics and early cancer screening: A concise review. Carbohydr. Polym..

[32] Kouisni L., Rochefort D. (2009). Confocal microscopy study of polymer microcapsules for enzyme immobilisation in paper substrates. J. Appl. Polym. Sci..

[33] Akyazi T., Basabe-Desmonts L., Benito-Lopez F. (2018). Review on microfluidic paper-based analytical devices towards commercialisation. Anal. Chim. Acta.

[34] Fu L.M., Wang Y.N. (2018). Detection methods and applications of microfluidic paper-based analytical devices. TrAC Trends Anal. Chem..

[35] Tang R.H., Yang H., Choi J.R., Gong Y., Feng S.S., Pingguan-Murphy B., Huang Q.S., Shi J.L., Mei Q.B., Xu F. (2017). Advances in paper-based sample pretreatment for point-of-care testing. Crit. Rev. Biotechnol..

[36] Bracher P.J., Gupta M., Whitesides G.M. (2010). Patterned paper as a template for the delivery of reactants in the fabrication of planar materials. Soft Matter.

[37] Tang R.H., Liu L.N., Zhang S.F., He X.C., Li X.J., Xu F., Ni Y.H., Li F. (2019). A review on advances in methods for modification of paper supports for use in point-of-care testing. Microchim. Acta.

[38] Bongiovanni R., Zeno E., Pollicino A., Serafini P.M., Tonelli C. (2011). UV light-induced grafting of fluorinated monomer onto cellulose sheets. Cellulose.

[39] Princi E., Vicini S. (2008). Graft polymerisation of ethyl acrylate/methyl methacrylate copolymers: A tool for the consolidation of paper-based materials. Eur. Polym. J..

[40] Liana D.D., Raguse B., Justin Gooding J., Chow E. (2012). Recent advances in paper-based sensors. Sensors.

[41] Kühl S., Krummenauer F., Dagassan-Berndt D., Lambrecht T.J., d’Hoedt B., Schulze R.K.W. (2011). Ink-jet printout of radiographs on transparent film and glossy paper versus monitor display: An ROC analysis. Clin. Oral Investig..

[42] Lin Y., Gritsenko D., Liu Q., Lu X., Xu J. (2016). Recent Advancements in Functionalized Paper-Based Electronics. ACS Appl. Mater. Interfaces.

[43] Kamel S., Khattab T.A. (2020). Recent advances in cellulose-based biosensors for medical diagnosis. Biosensors.

[44] Li Z., Askim J.R., Suslick K.S. (2019). The Optoelectronic Nose: Colorimetric and Fluorometric Sensor Arrays. Chem. Rev..

[45] Li Z., Suslick K.S. (2021). The Optoelectronic Nose. Acc. Chem. Res..

[46] Sola Martínez R.A., Pastor Hernández J.M., Yanes Torrado Ó., Cánovas Díaz M., de Diego Puente T., Vinaixa Crevillent M. (2021). Exhaled volatile organic compounds analysis in clinical pediatrics: A systematic review. Pediatr. Res..

[47] Oakley-Girvan I., Davis S.W. (2017). Breath based volatile organic compounds in the detection of breast, lung, and colorectal cancers: A systematic review. Cancer Biomark..

[48] Bel’skaya L.V., Sarf E.A., Shalygin S.P., Postnova T.V., Kosenok V.K. (2020). Identification of salivary volatile organic compounds as potential markers of stomach and colorectal cancer: A pilot study. J. Oral Biosci..

[49] Mirzaei Y., Gholami A., Bordbar M.M. (2021). A distance-based paper sensor for rapid detection of blood lactate concentration using gold nanoparticles synthesized by Satureja hortensis. Sens. Actuators B Chem..

[50] Khalid T., Aggio R., White P., De Lacy Costello B., Persad R., Al-Kateb H., Jones P., Probert C.S., Ratcliffe N. (2015). Urinary volatile organic compounds for the detection of prostate cancer. PLoS ONE.

[51] Roy M., Yadav B.K. (2021). Electronic nose for detection of food adulteration: A review. J. Food Sci. Technol..

[52] Bordbar M.M., Tashkhourian J., Hemmateenejad B. (2018). Qualitative and quantitative analysis of toxic materials in adulterated fruit pickle samples by a colorimetric sensor array. Sens. Actuators B Chem..

[53] Hemmateenejad B., Tashkhourian J., Bordbar M.M., Mobaraki N. (2017). Development of colorimetric sensor array for discrimination of herbal medicine. J. Iran. Chem. Soc..

[54] Shi H., Zhang M., Adhikari B. (2018). Advances of electronic nose and its application in fresh foods: A review. Crit. Rev. Food Sci. Nutr..

[55] Zhang Y., Askim J.R., Zhong W., Orlean P., Suslick K.S. (2014). Identification of pathogenic fungi with an optoelectronic nose. Analyst.

[56] Lough F., Perry J.D., Stanforth S.P., Dean J.R. (2017). Detection of exogenous VOCs as a novel in vitro diagnostic technique for the detection of pathogenic bacteria. TrAC Trends Anal. Chem..

[57] Tai H., Duan Z., Wang Y., Wang S., Jiang Y. (2020). Paper-Based Sensors for Gas, Humidity, and Strain Detections: A Review. ACS Appl. Mater. Interfaces.

[58] Boeker P. (2014). On ‘electronic nose’methodology. Sens. Actuators B Chem..

[59] Morbioli G.G., Mazzu-Nascimento T., Stockton A.M., Carrilho E. (2017). Technical aspects and challenges of colorimetric detection with microfluidic paper-based analytical devices (μPADs)—A review. Anal. Chim. Acta.

[60] He Y., Wu Y., Fu J.Z., Wu W. (2015). Bin Fabrication of paper-based microfluidic analysis devices: A review. RSC Adv..

[61] Podrazka M., Báczyńska E., Kundys M., Jeleń P.S., Nery E.W. (2017). Electronic tongue-A tool for all tastes?. Biosensors.

[62] Sharifi H., Tashkhourian J., Hemmateenejad B. (2020). A 3D origami paper-based analytical device combined with PVC membrane for colorimetric assay of heavy metal ions: Application to determination of Cu(II) in water samples. Anal. Chim. Acta.

[63] Zhang Y., Li X., Li H., Song M., Feng L., Guan Y. (2014). Postage stamp-sized array sensor for the sensitive screening test of heavy-metal ions. Analyst.

[64] Yetisen A.K., Akram M.S., Lowe C.R. (2013). Paper-based microfluidic point-of-care diagnostic devices. Lab Chip.

[65] Nguyen Q.H., Kim M. (2020). Il Nanomaterial-mediated paper-based biosensors for colorimetric pathogen detection. TrAC Trends Anal. Chem..

[66] Quesada-González D., Merkoçi A. (2015). Nanoparticle-based lateral flow biosensors. Biosens. Bioelectron..

[67] Parolo C., Sena-Torralba A., Bergua J.F., Calucho E., Fuentes-Chust C., Hu L., Rivas L., Álvarez-Diduk R., Nguyen E.P., Cinti S. (2020). Tutorial: Design and fabrication of nanoparticle-based lateral-flow immunoassays. Nat. Protoc..

[68] Chen Y., Chen Q., Han M., Zhou J., Gong L., Niu Y., Zhang Y., He L., Zhang L. (2016). Development and optimization of a multiplex lateral flow immunoassay for the simultaneous determination of three mycotoxins in corn, rice and peanut. Food Chem..

[69] Quesada-González D., Baiocco A., Martos A.A., de la Escosura-Muñiz A., Palleschi G., Merkoçi A. (2019). Iridium oxide (IV) nanoparticle-based electrocatalytic detection of PBDE. Biosens. Bioelectron..

[70] Tsai T.T., Huang T.H., Chen C.A., Ho N.Y.J., Chou Y.J., Chen C.F. (2018). Development a stacking pad design for enhancing the sensitivity of lateral flow immunoassay. Sci. Rep..

[71] Jia L., David M., Joanne M. (2015). Enhancing the signal of lateral flow immunoassays by using different developing methods. Sens. Mater..

[72] Bahadır E.B., Sezgintürk M.K. (2016). Lateral flow assays: Principles, designs and labels. TrAC Trends Anal. Chem..

[73] Luo K., Kim H.Y., Oh M.H., Kim Y.R. (2020). Paper-based lateral flow strip assay for the detection of foodborne pathogens: Principles, applications, technological challenges and opportunities. Crit. Rev. Food Sci. Nutr..

[74] Huang Y., Xu T., Wang W., Wen Y., Li K., Qian L., Zhang X., Liu G. (2020). Lateral flow biosensors based on the use of micro- and nanomaterials: A review on recent developments. Microchim. Acta.

[75] Sharma R., Gautam P.B., Rajput Y.S., Mann B., Gandhi K. (2019). Identification of analyte of interest through lateral flow assay. In Nano-Technological and Biochemical Techniques for Assessing the Quality and Safety of Milk and Milk Products. https://www.academia.edu/download/58315915/CAFT_Compendium.pdf#page=111.

[76] Byzova N.A., Zherdev A.V., Khlebtsov B.N., Burov A.M., Khlebtsov N.G., Dzantiev B.B. (2020). Advantages of highly spherical gold nanoparticles as labels for lateral flow immunoassay. Sensors.

[77] Nguyen V.T., Song S., Park S., Joo C. (2020). Recent advances in high-sensitivity detection methods for paper-based lateral-flow assay. Biosens. Bioelectron..

[78] Apilux A., Rengpipat S., Suwanjang W., Chailapakul O. (2018). Development of competitive lateral flow immunoassay coupled with silver enhancement for simple and sensitive salivary cortisol detection. EXCLI J..

[79] Rey E.G., O’Dell D., Mehta S., Erickson D. (2017). Mitigating the Hook Effect in Lateral Flow Sandwich Immunoassays Using Real-Time Reaction Kinetics. Anal. Chem..

[80] Ge X., Asiri A.M., Du D., Wen W., Wang S., Lin Y. (2014). Nanomaterial-enhanced paper-based biosensors. TrAC Trends Anal. Chem..

[81] Ross G.M.S., Salentijn G.I., Nielen M.W.F. (2019). A critical comparison between flow-through and lateral flow immunoassay formats for visual and smartphone-based multiplex allergen detection. Biosensors.

[82] Taghizadeh-Behbahani M., Hemmateenejad B., Shamsipur M., Tavassoli A. (2019). A paper-based length of stain analytical device for naked eye (readout-free) detection of cystic fibrosis. Anal. Chim. Acta.

[83] Alsaeed B., Mansour F.R. (2020). Distance-based paper microfluidics; principle, technical aspects and applications. Microchem. J..

[84] Zhang J.X.J., Hoshino K. (2019). Microfluidics and micro total analytical systems. Molecular Sensors and Nanodevices: Principles, Designs and Applications in Biomedical Engineering.

[85] Jiang L., Korivi N.S. (2013). Microfluidics: Technologies and applications. Nanolithogr. Art Fabr. Nanoelectron. Nanophotonic Devices Syst..

[86] Melin J., Van Der Wijngaart W., Stemme G. (2005). Behaviour and design considerations for continuous flow closed-open-closed liquid microchannels. Lab Chip.

[87] Konda A., Morin S.A. (2017). Flow-directed synthesis of spatially variant arrays of branched zinc oxide mesostructures. Nanoscale.

[88] Chokkalingam V., Tel J., Wimmers F., Liu X., Semenov S., Thiele J., Figdor C.G., Huck W.T.S. (2013). Probing cellular heterogeneity in cytokine-secreting immune cells using droplet-based microfluidics. Lab Chip.

[89] Pesant J., Hareng M., Mourey B., Perbet J. (1986). Electrodes for a Device Operating by Electrically Controlled Fluid Displacement. U.S. Patent.

[90] Martinez A.W., Phillips S.T., Butte M.J., Whitesides G.M. (2007). Patterned Paper as a Platform for Inexpensive, Low-Volume, Portable Bioassays. Angew. Chem..

[91] DeBlois R.W., Bean C.P. (1970). Counting and sizing of submicron particles by the resistive pulse technique. Rev. Sci. Instrum..

[92] Kauffman P., Fu E., Lutz B., Yager P. (2010). Visualization and measurement of flow in two-dimensional paper networks. Lab Chip.

[93] Martinez A.W., Phillips S.T., Whitesides G.M. (2008). Three-dimensional microfluidic devices fabricated in layered paper and tape. Proc. Natl. Acad. Sci. USA.

[94] Liu H., Crooks R.M. (2011). Three-dimensional paper microfluidic devices assembled using the principles of origami. J. Am. Chem. Soc..

[95] Santhiago M., Nery E.W., Santos G.P., Kubota L.T. (2014). Microfluidic paper-based devices for bioanalytical applications. Bioanalysis.

[96] Lim H., Jafry A.T., Lee J. (2019). Fabrication, flow control, and applications of microfluidic paper-based analytical devices. Molecules.

[97] Fenton E.M., Mascarenas M.R., López G.P., Sibbett S.S. (2009). Multiplex lateral-flow test strips fabricated by two-dimensional shaping. ACS Appl. Mater. Interfaces.

[98] Sadri B., Goswami D., Martinez R.V. (2018). Rapid fabrication of epidermal paper-based electronic devices using razor printing. Micromachines.

[99] Cassano C.L., Fan Z.H. (2013). Laminated paper-based analytical devices (LPAD): Fabrication, characterization, and assays. Microfluid. Nanofluidics.

[100] Jafry A.T., Lim H., Sung W.K., Lee J. (2017). Flexible time–temperature indicator: A versatile platform for laminated paper-based analytical devices. Microfluid. Nanofluidics.

[101] Glavan A.C., Martinez R.V., Maxwell E.J., Subramaniam A.B., Nunes R.M.D., Soh S., Whitesides G.M. (2013). Rapid fabrication of pressure-driven open-channel microfluidic devices in omniphobic RF paper. Lab Chip.

[102] Thuo M.M., Martinez R.V., Lan W.J., Liu X., Barber J., Atkinson M.B.J., Bandarage D., Bloch J.F., Whitesides G.M. (2014). Fabrication of low-cost paper-based microfluidic devices by embossing or cut-and-stack methods. Chem. Mater..

[103] Theillet G., Rubens A., Foucault F., Dalbon P., Rozand C., Leparc-Goffart I., Bedin F. (2018). Laser-cut paper-based device for the detection of dengue non-structural NS1 protein and specific IgM in human samples. Arch. Virol..

[104] Spicar-Mihalic P., Houghtaling J., Fu E., Yager P., Liang T., Toley B. (2013). CO_2_ laser cutting and ablative etching for the fabrication of paper-based devices. J. Micromech. Microeng..

[105] Xia Y., Si J., Li Z. (2016). Fabrication techniques for microfluidic paper-based analytical devices and their applications for biological testing: A review. Biosens. Bioelectron..

[106] Jeong S.G., Kim J., Nam J.O., Song Y.S., Lee C.S. (2013). Paper-based analytical device for quantitative urinalysis. Int. Neurourol. J..

[107] Songok J., Tuominen M., Teisala H., Haapanen J., Mäkelä J.M., Kuusipalo J., Toivakka M. (2016). Paper-Based Microfluidics: Fabrication Technique and Dynamics of Capillary-Driven Surface Flow. ACS Appl. Mater. Interfaces.

[108] Sones C.L., Katis I.N., He P.J.W., Mills B., Namiq M.F., Shardlow P., Ibsen M., Eason R.W. (2014). Laser-induced photo-polymerisation for creation of paper-based fluidic devices. Lab Chip.

[109] Nargang T.M., Dierkes R., Bruchmann J., Keller N., Sachsenheimer K., Lee-Thedieck C., Kotz F., Helmer D., Rapp B.E. (2018). Photolithographic structuring of soft, extremely foldable and autoclavable hydrophobic barriers in paper. Anal. Methods.

[110] Carrilho E., Martinez A.W., Whitesides G.M. (2009). Understanding wax printing: A simple micropatterning process for paper-based microfluidics. Anal. Chem..

[111] Suresh V., Qunya O., Kanta B.L., Yuh L.Y., Chong K.S.L. (2018). Non-invasive paper-based microfluidic device for ultra-low detection of urea through enzyme catalysis. R. Soc. Open Sci..

[112] Songjaroen T., Dungchai W., Chailapakul O., Laiwattanapaisal W. (2011). Novel, simple and low-cost alternative method for fabrication of paper-based microfluidics by wax dipping. Talanta.

[113] Zhang A.L., Zha Y. (2012). Fabrication of paper-based microfluidic device using printed circuit technology. AIP Adv..

[114] Noh H., Phillips S.T. (2010). Metering the capillary-driven flow of fluids in paper-based microfluidic devices. Anal. Chem..

[115] Maejima K., Tomikawa S., Suzuki K., Citterio D. (2013). Inkjet printing: An integrated and green chemical approach to microfluidic paper-based analytical devices. RSC Adv..

[116] Li X., Tian J., Garnier G., Shen W. (2010). Fabrication of paper-based microfluidic sensors by printing. Colloids Surf. B Biointerfaces.

[117] Wang J., Monton M.R.N., Zhang X., Filipe C.D.M., Pelton R., Brennan J.D. (2014). Hydrophobic sol-gel channel patterning strategies for paper-based microfluidics. Lab Chip.

[118] Hiltunen J., Liedert C., Hiltunen M., Huttunen O.H., Hiitola-Keinänen J., Aikio S., Harjanne M., Kurkinen M., Hakalahti L., Lee L.P. (2018). Roll-to-roll fabrication of integrated PDMS-paper microfluidics for nucleic acid amplification. Lab Chip.

[119] Olkkonen J., Lehtinen K., Erho T. (2010). Flexographically printed fluidic structures in paper. Anal. Chem..

[120] Bracher P.J., Gupta M., MacK E.T., Whitesides G.M. (2009). Heterogeneous films of ionotropic hydrogels fabricated from delivery templates of patterned paper. ACS Appl. Mater. Interfaces.

[121] Ghosh R., Gopalakrishnan S., Savitha R., Renganathan T., Pushpavanam S. (2019). Fabrication of laser printed microfluidic paper-based analytical devices (LP-µPADs) for point-of-care applications. Sci. Rep..

[122] Mohamed H.M. (2016). Screen-printed disposable electrodes: Pharmaceutical applications and recent developments. TrAC Trends Anal. Chem..

[123] Lamas-Ardisana P.J., Martínez-Paredes G., Añorga L., Grande H.J. (2018). Glucose biosensor based on disposable electrochemical paper-based transducers fully fabricated by screen-printing. Biosens. Bioelectron..

[124] Beitollahi H., Mohammadi S.Z., Safaei M., Tajik S. (2020). Applications of electrochemical sensors and biosensors based on modified screen-printed electrodes: A review. Anal. Methods.

[125] He Y., Wu Y., Fu J.Z., Gao Q., Qiu J.J. (2016). Developments of 3D Printing Microfluidics and Applications in Chemistry and Biology: A Review. Electroanalysis.

[126] He Y., Gao Q., Wu W.B., Nie J., Fu J.Z. (2016). 3D printed paper-based microfluidic analytical devices. Micromachines.

[127] Curto V.F., Lopez-Ruiz N., Capitan-Vallvey L.F., Palma A.J., Benito-Lopez F., Diamond D. (2013). Fast prototyping of paper-based microfluidic devices by contact stamping using indelible ink. RSC Adv..

[128] Yao X.H., Jia T., Xie C.Q., Fu J.Z., He Y. (2017). Facial fabrication of paper-based flexible electronics with flash foam stamp lithography. Microsyst. Technol..

[129] Liu N., Xu J., An H.J., Phan D.T., Hashimoto M., Lew W.S. (2017). Direct spraying method for fabrication of paper-based microfluidic devices. J. Micromech. Microeng..

[130] Nurak T., Praphairaksit N., Chailapakul O. (2013). Fabrication of paper-based devices by lacquer spraying method for the determination of nickel (II) ion in waste water. Talanta.

[131] Kwong P., Gupta M. (2012). Vapor phase deposition of functional polymers onto paper-based microfluidic devices for advanced unit operations. Anal. Chem..

[132] Lawal A.T., Wallace G.G. (2014). Vapour phase polymerisation of conducting and non-conducting polymers: A review. Talanta.

[133] Demirel G., Babur E. (2014). Vapor-phase deposition of polymers as a simple and versatile technique to generate paper-based microfluidic platforms for bioassay applications. Analyst.

[134] Obeso C.G., Sousa M.P., Song W., Rodriguez-Pérez M.A., Bhushan B., Mano J.F. (2013). Modification of paper using polyhydroxybutyrate to obtain biomimetic superhydrophobic substrates. Colloids Surf. A Physicochem. Eng. Asp..

[135] Sheini A., Aseman M.D., Bordbar M.M. (2021). Origami paper analytical assay based on metal complex sensor for rapid determination of blood cyanide concentration in fire survivors. Sci. Rep..

[136] Sheini A. (2020). A paper-based device for the colorimetric determination of ammonia and carbon dioxide using thiomalic acid and maltol functionalized silver nanoparticles: Application to the enzymatic determination of urea in saliva and blood. Microchim. Acta.

[137] Jeong S.G., Lee S.H., Choi C.H., Kim J., Lee C.S. (2015). Toward instrument-free digital measurements: A three-dimensional microfluidic device fabricated in a single sheet of paper by double-sided printing and lamination. Lab Chip.

[138] Mohammadifar M., Zhang J., Yazgan I., Sadik O., Choi S. (2018). Power-on-paper: Origami-inspired fabrication of 3-D microbial fuel cells. Renew. Energy.

[139] Park C., Han Y.D., Kim H.V., Lee J., Yoon H.C., Park S. (2018). Double-sided 3D printing on paper towards mass production of three-dimensional paper-based microfluidic analytical devices (3D-μPADs). Lab Chip.

[140] Ding J., Li B., Chen L., Qin W. (2016). A Three-Dimensional Origami Paper-Based Device for Potentiometric Biosensing. Angew. Chem. Int. Ed..

[141] Razmi N., Baradaran B., Hejazi M., Hasanzadeh M., Mosafer J., Mokhtarzadeh A., de la Guardia M. (2018). Recent advances on aptamer-based biosensors to detection of platelet-derived growth factor. Biosens. Bioelectron..

[142] Juzgado A., Soldà A., Ostric A., Criado A., Valenti G., Rapino S., Conti G., Fracasso G., Paolucci F., Prato M. (2017). Highly sensitive electrochemiluminescence detection of a prostate cancer biomarker. J. Mater. Chem. B.

[143] Vo-Dinh T., Cullum B. (2000). Biosensors and biochips: Advances in biological and medical diagnostics. Fresenius. J. Anal. Chem..

[144] Valenti G., Rampazzo E., Biavardi E., Villani E., Fracasso G., Marcaccio M., Bertani F., Ramarli D., Dalcanale E., Paolucci F. (2015). An electrochemiluminescence-supramolecular approach to sarcosine detection for early diagnosis of prostate cancer. Faraday Discuss..

[145] Holford T.R.J., Davis F., Higson S.P.J. (2012). Recent trends in antibody based sensors. Biosens. Bioelectron..

[146] Aleman J., Kilic T., Mille L.S., Shin S.R., Zhang Y.S. (2021). Microfluidic integration of regeneratable electrochemical affinity-based biosensors for continual monitoring of organ-on-a-chip devices. Nat. Protoc..

[147] JD B., BG B., WG V. (1987). Measurement of monoclonal antibody affinity by non-competitive enzyme immunoassay. J. Immunol. Methods.

[148] Nistor C., Emnéus J. (2005). Chapter 9 Immunoassay: Potentials and limitations. Compr. Anal. Chem..

[149] Jost C., Plückthun A. (2014). Engineered proteins with desired specificity: DARPins, other alternative scaffolds and bispecific IgGs. Curr. Opin. Struct. Biol..

[150] Mehlhorn A., Rahimi P., Joseph Y. (2018). Aptamer-based biosensors for antibiotic detection: A review. Biosensors.

[151] Mello L.D., Kubota L.T. (2002). Review of the use of biosensors as analytical tools in the food and drink industries. Food Chem..

[152] Du Y., Dong S. (2017). Nucleic acid biosensors: Recent advances and perspectives. Anal. Chem..

[153] Bollella P., Gorton L. (2018). Enzyme based amperometric biosensors. Curr. Opin. Electrochem..

[154] Mross S., Pierrat S., Zimmermann T., Kraft M. (2015). Microfluidic enzymatic biosensing systems: A review. Biosens. Bioelectron..

[155] Marazuela M.D., Moreno-Bondi M.C. (2002). Fiber-optic biosensors—An overview. Anal. Bioanal. Chem..

[156] Upadhyay L., Verm N. (2013). Enzyme Inhibition Based Biosensors: A Review. Anal. Lett..

[157] Mehrotra P. (2016). Biosensors and their applications—A review. J. Oral Biol. Craniofacial Res..

[158] Kazemi-Darsanaki R., Azizzadeh A., Nourbakhsh M., Raeisi G., AzizollahiAliabadi M. (2013). Biosensors: Functions and Applications. J. Biol. Today’s World.

[159] Nakamura H. (2018). Current status of water environment and their microbial biosensor techniques—Part II: Recent trends in microbial biosensor development. Anal. Bioanal. Chem..

[160] Varzakas T., Nikoleli G.-P., Nikolelis D. (2013). Tissue, Microorganisms, Organelles, and Cell-Based Biosensors.

[161] Campàs M., Carpentier R., Rouillon R. (2008). Plant tissue-and photosynthesis-based biosensors. Biotechnol. Adv..

[162] Jung Y., Jeong J.Y., Chung B.H. (2008). Recent advances in immobilization methods of antibodies on solid supports. Analyst.

[163] Nery E.W., Kubota L.T. (2016). Evaluation of enzyme immobilization methods for paper-based devices—A glucose oxidase study. J. Pharm. Biomed. Anal..

[164] Kong F., Hu Y.F. (2012). Biomolecule immobilization techniques for bioactive paper fabrication. Anal. Bioanal. Chem..

[165] Narsaiah K., Jha S.N., Bhardwaj R., Sharma R., Kumar R. (2012). Optical biosensors for food quality and safety assurance—A review. J. Food Sci. Technol..

[166] Kaur K., Kaushal P. (2019). Enzymes as analytical tools for the assessment of food quality and food safety. Biomass Biofuels Biochem. Adv. Enzym. Technol..

[167] Yamaguchi H., Kiyota Y., Miyazaki M. (2018). Techniques for preparation of cross-linked enzyme aggregates and their applications in bioconversions. Catalysts.

[168] Liebich V.J., Avrutina O., Habermann J., Hillscher L.M., Langhans M., Meckel T., Biesalski M., Kolmar H. (2021). Toward Fabrication of Bioactive Papers: Covalent Immobilization of Peptides and Proteins. Biomacromolecules.

[169] Kasoju N., Nguyen L.T.B., Padalhin A.R., Dye J.F., Cui Z., Ye H. (2018). Techniques for modifying biomaterials to improve hemocompatibility. Hemocompat. Biomater. Clin. Appl. Blood-Biomater. Interact..

[170] Al-Husseini Z.N.O. (2019). A Literature Review on the Indicators in Precipitation. Am. Int. J. Sci. Eng. Res..

[171] Martinez A.W., Phillips S.T., Whitesides G.M., Carrilho E. (2010). Diagnostics for the developing world: Microfluidic paper-based analytical devices. Anal. Chem..

[172] Liu M.M., Lian X., Liu H., Guo Z.Z., Huang H.H., Lei Y., Peng H.P., Chen W., Lin X.H., Liu A.L. (2019). A colorimetric assay for sensitive detection of hydrogen peroxide and glucose in microfluidic paper-based analytical devices integrated with starch-iodide-gelatin system. Talanta.

[173] Almeida L.C., Correia J.P., Viana A.S. (2018). Electrochemical and optical characterization of thin polydopamine films on carbon surfaces for enzymatic sensors. Electrochim. Acta.

[174] Mu C., Lu H., Bao J., Zhang Q. (2018). Visual colorimetric ‘turn-off’ biosensor for ascorbic acid detection based on hypochlorite–3,3′,5,5′,-Tetramethylbenzidine system. Spectrochim. Acta Part A Mol. Biomol. Spectrosc..

[175] Gao S., Zheng X., Hu B., Sun M., Wu J., Jiao B., Wang L. (2017). Enzyme-linked, aptamer-based, competitive biolayer interferometry biosensor for palytoxin. Biosens. Bioelectron..

[176] Ding L., Gong Z., Yan M., Yu J., Song X. (2017). Determination of glucose by using fluorescent silicon nanoparticles and an inner filter caused by peroxidase-induced oxidation of o-phenylenediamine by hydrogen peroxide. Microchim. Acta.

[177] Fan Y., Shi S., Ma J., Guo Y. (2019). A paper-based electrochemical immunosensor with reduced graphene oxide/thionine/gold nanoparticles nanocomposites modification for the detection of cancer antigen 125. Biosens. Bioelectron..

[178] Dutta G., Lillehoj P.B. (2017). An ultrasensitive enzyme-free electrochemical immunosensor based on redox cycling amplification using methylene blue. Analyst.

[179] Hamidi-Asl E., Raoof J.B., Ojani R., Hejazi M.S. (2013). Indigo carmine as new label in PNA biosensor for detection of short sequence of p53 tumor suppressor gene. Electroanalysis.

[180] Chen Q., Huang F., Cai G., Wang M., Lin J. (2018). An optical biosensor using immunomagnetic separation, urease catalysis and pH indication for rapid and sensitive detection of Listeria monocytogenes. Sens. Actuators B Chem..

[181] Van der Schueren L., de Clerck K. (2012). Coloration and application of pH-sensitive dyes on textile materials. Color. Technol..

[182] Crosland M., Hannaway O. (1981). Gay-Lussac, Scientist and Bourgeois. Phys. Today.

[183] Mahmoudi M., Lohse S.E., Murphy C.J., Suslick K.S. (2016). Identification of Nanoparticles with a Colorimetric Sensor Array. ACS Sens..

[184] Askim J.R., Mahmoudi M., Suslick K.S. (2013). Optical sensor arrays for chemical sensing: The optoelectronic nose. Chem. Soc. Rev..

[185] Sedgwick A.C., Brewster J.T., Wu T., Feng X., Bull S.D., Qian X., Sessler J.L., James T.D., Anslyn E.V., Sun X. (2021). Indicator displacement assays (IDAs): The past, present and future. Chem. Soc. Rev..

[186] Khajehsharifi H., Bordbar M.M. (2015). A highly selective chemosensor for detection and determination of cyanide by using an indicator displacement assay and PC-ANN and its logic gate behavior. Sens. Actuators B Chem..

[187] Ealia S.A.M., Saravanakumar M.P. (2017). A review on the classification, characterisation, synthesis of nanoparticles and their application. IOP Conf. Ser. Mater. Sci..

[188] Cuenya B.R. (2010). Synthesis and catalytic properties of metal nanoparticles: Size, shape, support, composition, and oxidation state effects. Thin Solid Film..

[189] Lan L., Yao Y., Ping J., Ying Y. (2017). Recent advances in nanomaterial-based biosensors for antibiotics detection. Biosens. Bioelectron..

[190] Zeng Y., Zhu Z., Du D., Lin Y. (2016). Nanomaterial-based electrochemical biosensors for food safety. J. Electroanal. Chem..

[191] Srikar S.K., Giri D.D., Pal D.B., Mishra P.K., Upadhyay S.N. (2016). Green Synthesis of Silver Nanoparticles: A Review. Green Sustain. Chem..

[192] Srinoi P., Chen Y.T., Vittur V., Marquez M.D., Lee T.R. (2018). Bimetallic nanoparticles: Enhanced magnetic and optical properties for emerging biological applications. Appl. Sci..

[193] Sharma G., Kumar A., Sharma S., Naushad M., Prakash Dwivedi R., ALOthman Z.A., Mola G.T. (2019). Novel development of nanoparticles to bimetallic nanoparticles and their composites: A review. J. King Saud Univ. Sci..

[194] Wu K., Su D., Liu J., Saha R., Wang J.P. (2019). Magnetic nanoparticles in nanomedicine: A review of recent advances. Nanotechnology.

[195] Pastucha M., Farka Z., Lacina K., Mikušová Z., Skládal P. (2019). Magnetic nanoparticles for smart electrochemical immunoassays: A review on recent developments. Microchim. Acta.

[196] Mohammed L., Gomaa H.G., Ragab D., Zhu J. (2017). Magnetic nanoparticles for environmental and biomedical applications: A review. Particuology.

[197] Asadian E., Ghalkhani M., Shahrokhian S. (2019). Electrochemical sensing based on carbon nanoparticles: A review. Sens. Actuators B Chem..

[198] LeCroy G.E., Yang S.T., Yang F., Liu Y., Fernando K.A.S., Bunker C.E., Hu Y., Luo P.G., Sun Y.P. (2016). Functionalized carbon nanoparticles: Syntheses and applications in optical bioimaging and energy conversion. Coord. Chem. Rev..

[199] Balarastaghi M., Ahmadi V. (2017). Formulation of atomic positions and carbon–carbon bond length in armchair graphene nanoribbons: An ab initio study. J. Theor. Appl. Phys..

[200] Ji K., Han J., Hirata A., Fujita T., Shen Y., Ning S., Liu P., Kashani H., Tian Y., Ito Y. (2019). Lithium intercalation into bilayer graphene. Nat. Commun..

[201] Kokorina A.A., Ermakov A.V., Abramova A.M., Goryacheva I.Y., Sukhorukov G.B. (2020). Carbon nanoparticles and materials on their basis. Colloids Interfaces.

[202] Anzar N., Hasan R., Tyagi M., Yadav N., Narang J. (2020). Carbon nanotube—A review on Synthesis, Properties and plethora of applications in the field of biomedical science. Sens. Int..

[203] Campuzano S., Yáñez-Sedeño P., Pingarrón J.M. (2019). Carbon dots and graphene quantum dots in electrochemical biosensing. Nanomaterials.

[204] Zhang Y., Zhang C., Xu C., Wang X., Liu C., Waterhouse G.I.N., Wang Y., Yin H. (2019). Ultrasmall Au nanoclusters for biomedical and biosensing applications: A mini-review. Talanta.

[205] Cui H., Shao Z.S., Song Z., Wang Y.B., Wang H.S. (2020). Development of gold nanoclusters: From preparation to applications in the field of biomedicine. J. Mater. Chem. C.

[206] Kimmel D.W., Leblanc G., Meschievitz M.E., Cliffel D.E. (2012). Electrochemical sensors and biosensors. Anal. Chem..

[207] Kanyong P., Krampa F.D., Aniweh Y., Awandare G.A. (2017). Enzyme-based amperometric galactose biosensors: A review. Microchim. Acta.

[208] Ding J., Qin W. (2020). Recent advances in potentiometric biosensors. TrAC Trends Anal. Chem..

[209] Bahadir E.B., Sezgintürk M.K. (2016). A review on impedimetric biosensors. Artif. Cells Nanomed. Biotechnol..

[210] Adley C.C., Ryan M.P. (2015). Conductometric biosensors for high throughput screening of pathogens in food. High Throughput Screen. Food Saf. Assess. Biosens. Technol. Hyperspectr. Imaging Pract. Appl..

[211] Chen D., Zhang M., Zhou F., Hai H., Li J. (2019). Ultrasensitive electroluminescence biosensor for a breast cancer marker microRNA based on target cyclic regeneration and multi-labeled magnetized nanoparticles. Microchim. Acta.

[212] Shamsi M.H., Choi K., Ng A.H.C., Dean Chamberlain M., Wheeler A.R. (2016). Electrochemiluminescence on digital microfluidics for microRNA analysis. Biosens. Bioelectron..

[213] Nie Z., Deiss F., Liu X., Akbulut O., Whitesides G.M. (2010). Integration of paper-based microfluidic devices with commercial electrochemical readers. Lab Chip.

[214] Millo T., Jaiswal A.K., Prasad Y.S., Murty O.P. (2010). Breath alcohol analyzer and its forensic applications. J. Forensic Med. Toxicol..

[215] Van Dyk J.S., Pletschke B. (2011). Review on the use of enzymes for the detection of organochlorine, organophosphate and carbamate pesticides in the environment. Chemosphere.

[216] Mercer C., Bennett R., Conghaile P., Rusling J.F., Leech D. (2019). Glucose biosensor based on open-source wireless microfluidic potentiostat. Sens. Actuators B Chem..

[217] Ramanathan K., Danielsson B. (2001). Principles and applications of thermal biosensors. Biosens. Bioelectron..

[218] Alan T. (2001). Improving the accuracy of temperature measurements. Sens. Rev..

[219] Yu L., Li N. (2019). Noble metal nanoparticles-based colorimetric biosensor for visual quantification: A mini review. Chemosensors.

[220] Huang X., Xu D., Chen J., Liu J., Li Y., Song J., Ma X., Guo J. (2018). Smartphone-based analytical biosensors. Analyst.

[221] Hemmateenejad B., Mobaraki N., Shakerizadeh-Shirazi F., Miri R. (2010). Multivariate image analysis-thin layer chromatography (MIA-TLC) for simultaneous determination of co-eluting components. Analyst.

[222] Leopold A.V., Shcherbakova D.M., Verkhusha V.V. (2019). Fluorescent Biosensors for Neurotransmission and Neuromodulation: Engineering and Applications. Front. Cell. Neurosci..

[223] Gaviria-Arroyave M.I., Cano J.B., Peñuela G.A. (2020). Nanomaterial-based fluorescent biosensors for monitoring environmental pollutants: A critical review. Talanta Open.

[224] Van De Weert M., Stella L. (2011). Fluorescence quenching and ligand binding: A critical discussion of a popular methodology. J. Mol. Struct..

[225] Ulep T.H., Yoon J.Y. (2018). Challenges in paper-based fluorogenic optical sensing with smartphones. Nano Converg..

[226] Biological Toxin Safe Work Practices. https://www.ehs.washington.edu/resource/biological-toxin-safe-work-practices-65.

[227] Singh J., Mehta A. (2020). Rapid and sensitive detection of mycotoxins by advanced and emerging analytical methods: A review. Food Sci. Nutr..

[228] Haque M.A., Wang Y., Shen Z., Li X., Saleemi M.K., He C. (2020). Mycotoxin contamination and control strategy in human, domestic animal and poultry: A review. Microb. Pathog..

[229] Al-Jaal B.A., Jaganjac M., Barcaru A., Horvatovich P., Latiff A. (2019). Aflatoxin, fumonisin, ochratoxin, zearalenone and deoxynivalenol biomarkers in human biological fluids: A systematic literature review, 2001–2018. Food Chem. Toxicol..

[230] Chauhan R., Singh J., Sachdev T., Basu T., Malhotra B.D. (2016). Recent advances in mycotoxins detection. Biosens. Bioelectron..

[231] Liu D., Li W., Zhu C., Li Y., Shen X., Li L., Yan X., You T. (2020). Recent progress on electrochemical biosensing of aflatoxins: A review. TrAC Trends Anal. Chem..

[232] Wannop C.C. (1961). The Histopathology of Turkey “X” Disease in Great Britain. Avian Dis..

[233] Negash D. (2018). A Review of Aflatoxin: Occurrence, Prevention, and Gaps in Both Food and Feed Safety. J. Appl. Microb. Res..

[234] Robertson A. Risk of Aflatoxin Contamination Increases with Hot and Dry Growing Conditions. https://lib.dr.iastate.edu/cropnews/1383/.

[235] FDA Guidance for Industry: Action Levels for Poisonous or Deleterious Substances in Human Food and Animal Feed. https://www.fda.gov/media/121202/download.

[236] Xue Z., Zhang Y., Yu W., Zhang J., Wang J., Wan F., Kim Y., Liu Y., Kou X. (2019). Recent advances in aflatoxin B1 detection based on nanotechnology and nanomaterials-A review. Anal. Chim. Acta.

[237] Liu D., Huang Y., Chen M., Wang S., Liu K., Lai W. (2015). Rapid detection method for aflatoxin B1 in soybean sauce based on fluorescent microspheres probe. Food Control.

[238] Li M., Wang H., Sun J., Ji J., Ye Y., Lu X., Zhang Y., Sun X. (2021). Rapid, on-site, and sensitive detection of aflatoxin M1 in milk products by using time-resolved fluorescence microsphere test strip. Food Control.

[239] Yang Q., Zhu J., Ma F., Li P., Zhang L., Zhang W., Ding X., Zhang Q. (2016). Quantitative determination of major capsaicinoids in serum by ELISA and time-resolved fluorescent immunoassay based on monoclonal antibodies. Biosens. Bioelectron..

[240] Tang X., Li P., Zhang Q., Zhang Z., Zhang W., Jiang J. (2017). Time-Resolved Fluorescence Immunochromatographic Assay Developed Using Two Idiotypic Nanobodies for Rapid, Quantitative, and Simultaneous Detection of Aflatoxin and Zearalenone in Maize and Its Products. Anal. Chem..

[241] Wang Q.X., Xue S.F., Chen Z.H., Ma S.H., Zhang S., Shi G., Zhang M. (2017). Dual lanthanide-doped complexes: The development of a time-resolved ratiometric fluorescent probe for anthrax biomarker and a paper-based visual sensor. Biosens. Bioelectron..

[242] Tang X., Zhang Q., Zhang Z., Ding X., Jiang J., Zhang W., Li P. (2019). Rapid, on-site and quantitative paper-based immunoassay platform for concurrent determination of pesticide residues and mycotoxins. Anal. Chim. Acta.

[243] Wang C., Peng J., Liu D.F., Xing K.Y., Zhang G.G., Huang Z., Cheng S., Zhu F.F., Duan M.L., Zhang K.Y. (2018). Lateral flow immunoassay integrated with competitive and sandwich models for the detection of aflatoxin M1 and Escherichia coli O157:H7 in milk. J. Dairy Sci..

[244] Li X., Li P., Zhang Q., Li R., Zhang W., Zhang Z., Ding X., Tang X. (2013). Multi-component immunochromatographic assay for simultaneous detection of aflatoxin B1, ochratoxin A and zearalenone in agro-food. Biosens. Bioelectron..

[245] Kong D., Liu L., Song S., Suryoprabowo S., Li A., Kuang H., Wang L., Xu C. (2016). A gold nanoparticle-based semi-quantitative and quantitative ultrasensitive paper sensor for the detection of twenty mycotoxins. Nanoscale.

[246] Wang Y., Liu N., Ning B., Liu M., Lv Z., Sun Z., Peng Y., Chen C., Li J., Gao Z. (2012). Simultaneous and rapid detection of six different mycotoxins using an immunochip. Biosens. Bioelectron..

[247] Zhang G., Zhu C., Huang Y., Yan J., Chen A. (2018). A lateral flow strip based aptasensor for detection of Ochratoxin a in corn samples. Molecules.

[248] Kyung-Mi S., Seonghwan L., Changill B. (2012). Aptamers and Their Biological Applications. Sensors.

[249] Kasoju A., Shahdeo D., Khan A.A., Shrikrishna N.S., Mahari S., Alanazi A.M., Bhat M.A., Giri J., Gandhi S. (2020). Fabrication of microfluidic device for Aflatoxin M1 detection in milk samples with specific aptamers. Sci. Rep..

[250] Sheini A. (2020). Colorimetric aggregation assay based on array of gold and silver nanoparticles for simultaneous analysis of aflatoxins, ochratoxin and zearalenone by using chemometric analysis and paper based analytical devices. Microchim. Acta.

[251] Migliorini F.L., dos Santos D.M., Soares A.C., Mattoso L.H.C., Oliveira O.N., Correa D.S. (2020). Design of a low-cost and disposable paper-based immunosensor for the rapid and sensitive detection of aflatoxin B1. Chemosensors.

[252] Ye Y., Zhou Y., Mo Z., Cheng W., Yang S., Wang X., Chen F. (2010). Rapid detection of aflatoxin B1 on membrane by dot-immunogold filtration assay. Talanta.

[253] Liao J.Y., Li H. (2010). Lateral flow immunodipstick for visual detection of aflatoxin B1 in food using immuno-nanoparticles composed of a silver core and a gold shell. Microchim. Acta.

[254] Kasoju A., Shrikrishna N.S., Shahdeo D., Khan A.A., Alanazi A.M., Gandhi S. (2020). Microfluidic paper device for rapid detection of aflatoxin B1 using an aptamer based colorimetric assay. RSC Adv..

[255] Song S., Liu N., Zhao Z., Njumbe Ediage E., Wu S., Sun C., De Saeger S., Wu A. (2014). Multiplex lateral flow immunoassay for mycotoxin determination. Anal. Chem..

[256] Pirsaheb M., Hossini H., Asadi F., Janjani H. (2017). A systematic review on organochlorine and organophosphorus pesticides content in water resources. Toxin Rev..

[257] Diauudin F.N., Rashid J.I.A., Knight V.F., Wan Yunus W.M.Z., Ong K.K., Kasim N.A.M., Abdul Halim N., Noor S.A.M. (2019). A review of current advances in the detection of organophosphorus chemical warfare agents based biosensor approaches. Sens. Bio-Sens. Res..

[258] Obare S.O., De C., Guo W., Haywood T.L., Samuels T.A., Adams C.P., Masika N.O., Murray D.H., Anderson G.A., Campbell K. (2010). Fluorescent chemosensors for toxic organophosphorus pesticides: A review. Sensors.

[259] Gourie-Devi M. (2006). Neurological practice: An Indian perspective. Ann. Indian Acad. Neurol..

[260] Hmouda H., Salem C.B., Bouraoui K. (2008). Management of acute organophosphorus pesticide poisoning. Lancet.

[261] Pundir C.S., Chauhan N. (2012). Acetylcholinesterase inhibition-based biosensors for pesticide determination: A review. Anal. Biochem..

[262] Kim H.J., Kim Y., Park S.J., Kwon C., Noh H. (2018). Development of Colorimetric Paper Sensor for Pesticide Detection Using Competitive-inhibiting Reaction. Biochip J..

[263] Fu Q., Zhang C., Xie J., Li Z., Qu L., Cai X., Ouyang H., Song Y., Du D., Lin Y. (2019). Ambient light sensor based colorimetric dipstick reader for rapid monitoring organophosphate pesticides on a smart phone. Anal. Chim. Acta.

[264] George J.M., Antony A., Mathew B. (2018). Metal oxide nanoparticles in electrochemical sensing and biosensing: A review. Microchim. Acta.

[265] Kargozar S., Baino F., Hoseini S.J., Hamzehlou S., Darroudi M., Verdi J., Hasanzadeh L., Kim H.W., Mozafari M. (2018). Biomedical applications of nanoceria: New roles for an old player. Nanomedicine.

[266] Liu B., Sun Z., Huang P.J.J., Liu J. (2015). Hydrogen peroxide displacing DNA from nanoceria: Mechanism and detection of glucose in serum. J. Am. Chem. Soc..

[267] Nouanthavong S., Nacapricha D., Henry C.S., Sameenoi Y. (2016). Pesticide analysis using nanoceria-coated paper-based devices as a detection platform. Analyst.

[268] Chang J., Li H., Hou T., Li F. (2016). Paper-based fluorescent sensor for rapid naked-eye detection of acetylcholinesterase activity and organophosphorus pesticides with high sensitivity and selectivity. Biosens. Bioelectron..

[269] Wang Q., Yin Q., Fan Y., Zhang L., Xu Y., Hu O., Guo X., Shi Q., Fu H., She Y. (2019). Double quantum dots-nanoporphyrin fluorescence-visualized paper-based sensors for detecting organophosphorus pesticides. Talanta.

[270] Xie J., Li L., Khan I.M., Wang Z., Ma X. (2020). Flexible paper-based SERS substrate strategy for rapid detection of methyl parathion on the surface of fruit. Spectrochim. Acta Part A Mol. Biomol. Spectrosc..

[271] Xiong S., Deng Y., Zhou Y., Gong D., Xu Y., Yang L., Chen H., Chen L., Song T., Luo A. (2018). Current progress in biosensors for organophosphorus pesticides based on enzyme functionalized nanostructures: A review. Anal. Methods.

[272] Arduini F., Cinti S., Caratelli V., Amendola L., Palleschi G., Moscone D. (2019). Origami multiple paper-based electrochemical biosensors for pesticide detection. Biosens. Bioelectron..

[273] Bigley A.N., Raushel F.M. (2013). Catalytic mechanisms for phosphotriesterases. Biochim. Biophys. Acta Proteins Proteom..

[274] Hondred J.A., Breger J.C., Alves N.J., Trammell S.A., Walper S.A., Medintz I.L., Claussen J.C. (2018). Printed Graphene Electrochemical Biosensors Fabricated by Inkjet Maskless Lithography for Rapid and Sensitive Detection of Organophosphates. ACS Appl. Mater. Interfaces.

[275] Mehta J., Vinayak P., Tuteja S.K., Chhabra V.A., Bhardwaj N., Paul A.K., Kim K.H., Deep A. (2016). Graphene modified screen printed immunosensor for highly sensitive detection of parathion. Biosens. Bioelectron..

[276] Bordbar M.M., Nguyen T.A., Arduini F., Bagheri H. (2020). A paper-based colorimetric sensor array for discrimination and simultaneous determination of organophosphate and carbamate pesticides in tap water, apple juice, and rice. Microchim. Acta.

[277] Bordbar M.M., Nguyen T.A., Tran A.Q., Bagheri H. (2020). Optoelectronic nose based on an origami paper sensor for selective detection of pesticide aerosols. Sci. Rep..

[278] Wang T., Reid R.C., Minteer S.D. (2016). A Paper-based Mitochondrial Electrochemical Biosensor for Pesticide Detection. Electroanalysis.

[279] Deng S., Yang T., Zhang W., Ren C., Zhang J., Zhang Y., Cui T., Yue W. (2019). Rapid detection of trichlorfon residues by a microfluidic paper-based phosphorus-detection chip (μPPC). New J. Chem..

[280] Yang N., Shaheen N., Xie L., Yu J., Ahmad H., Mao H. (2019). Pesticide residues identification by optical spectrum in the time-sequence of enzyme inhibitors performed on microfluidic paper-based analytical devices (μPADs). Molecules.

[281] Bridle H., Miller B., Desmulliez M.P.Y. (2014). Application of microfluidics in waterborne pathogen monitoring: A review. Water Res..

[282] Bordbar M.M., Tashkhourian J., Tavassoli A., Bahramali E., Hemmateenejad B. (2020). Ultrafast detection of infectious bacteria using optoelectronic nose based on metallic nanoparticles. Sens. Actuators B Chem..

[283] Gregersen T. (1978). Rapid method for distinction of gram-negative from gram-positive bacteria. Eur. J. Appl. Microbiol. Biotechnol..

[284] Saravanan A., Kumar P.S., Hemavathy R.V., Jeevanantham S., Kamalesh R., Sneha S., Yaashikaa P.R. (2021). Methods of detection of food-borne pathogens: A review. Environ. Chem. Lett..

[285] Rajapaksha P., Elbourne A., Gangadoo S., Brown R., Cozzolino D., Chapman J. (2019). A review of methods for the detection of pathogenic microorganisms. Analyst.

[286] Creran B., Li X., Duncan B., Kim C.S., Moyano D.F., Rotello V.M. (2014). Detection of bacteria using inkjet-printed enzymatic test strips. ACS Appl. Mater. Interfaces.

[287] Jokerst J.C., Adkins J.A., Bisha B., Mentele M.M., Goodridge L.D., Henry C.S. (2012). Development of a paper-based analytical device for colorimetric detection of select foodborne pathogens. Anal. Chem..

[288] Suaifan G.A.R.Y., Alhogail S., Zourob M. (2017). Rapid and low-cost biosensor for the detection of *Staphylococcus aureus*. Biosens. Bioelectron..

[289] Sun L., Jiang Y., Pan R., Li M., Wang R., Chen S., Fu S., Man C. (2018). A novel, simple and low-cost paper-based analytical device for colorimetric detection of *Cronobacter* spp.. Anal. Chim. Acta.

[290] Bagheri Pebdeni A., Hosseini M. (2020). Fast and selective whole cell detection of *Staphylococcus aureus* bacteria in food samples by paper based colorimetric nanobiosensor using peroxidase-like catalytic activity of DNA-Au/Pt bimetallic nanoclusters. Microchem. J..

[291] Wang Y., Ping J., Ye Z., Wu J., Ying Y. (2013). Impedimetric immunosensor based on gold nanoparticles modified graphene paper for label-free detection of Escherichia coli O157: H7. Biosens. Bioelectron..

[292] Mo X., Wu Z., Huang J., Zhao G., Dou W. (2019). A sensitive and regenerative electrochemical immunosensor for quantitative detection of: *Escherichia coli* O157:H7 based on stable polyaniline coated screen-printed carbon electrode and rGO-NR-Au@Pt. Anal. Methods.

[293] Khan M.S., Misra S.K., Dighe K., Wang Z., Schwartz-Duval A.S., Sar D., Pan D. (2018). Electrically-receptive and thermally-responsive paper-based sensor chip for rapid detection of bacterial cells. Biosens. Bioelectron..

[294] Hernández R., Vallés C., Benito A.M., Maser W.K., Xavier Rius F., Riu J. (2014). Graphene-based potentiometric biosensor for the immediate detection of living bacteria. Biosens. Bioelectron..

[295] Bhardwaj J., Devarakonda S., Kumar S., Jang J. (2017). Development of a paper-based electrochemical immunosensor using an antibody-single walled carbon nanotubes bio-conjugate modified electrode for label-free detection of foodborne pathogens. Sens. Actuators B Chem..

[296] Han J., Cheng H., Wang B., Braun M.S., Fan X., Bender M., Huang W., Domhan C., Mier W., Lindner T. (2017). A Polymer/Peptide Complex-Based Sensor Array That Discriminates Bacteria in Urine. Angew. Chem..

[297] Yan P., Ding Z., Li X., Dong Y., Fu T., Wu Y. (2019). Colorimetric Sensor Array Based on Wulff-Type Boronate Functionalized AgNPs at Various pH for Bacteria Identification. Anal. Chem..

[298] Sun H., Tian F., Liang Z., Sun T., Yu B., Yang S.X., He Q., Zhang L., Liu X. (2017). Sensor Array Optimization of Electronic Nose for Detection of Bacteria in Wound Infection. IEEE Trans. Ind. Electron..

[299] Wu Y., Wang B., Wang K., Yan P. (2018). Identification of proteins and bacteria based on a metal ion-gold nanocluster sensor array. Anal. Methods.

[300] Svechkarev D., Sadykov M.R., Bayles K.W., Mohs A.M. (2018). Ratiometric Fluorescent Sensor Array as a Versatile Tool for Bacterial Pathogen Identification and Analysis. ACS Sens..

[301] Lai S.Y., Deffenderfer O.F., Hanson W., Phillips M.P., Thaler E.R. (2002). Identification of upper respiratory bacterial pathogens with the electronic nose. Laryngoscope.

[302] Carey J.R., Suslick K.S., Hulkower K.I., Imlay J.A., Imlay K.R.C., Ingison C.K., Ponder J.B., Sen A., Wittrig A.E. (2011). Rapid identification of bacteria with a disposable colorimetric sensing array. J. Am. Chem. Soc..

[303] Lim S.H., Mix S., Xu Z., Taba B., Budvytiene I., Berliner A.N., Queralto N., Churi Y.S., Huang R.S., Eiden M. (2014). Colorimetric sensor array allows fast detection and simultaneous identification of sepsis-causing bacteria in spiked blood culture. J. Clin. Microbiol..

[304] Canhoto O., Magan N. (2005). Electronic nose technology for the detection of microbial and chemical contamination of potable water. Sens. Actuators B Chem..

[305] Dutta R., Das A., Stocks N.G., Morgan D. (2006). Stochastic resonance-based electronic nose: A novel way to classify bacteria. Sens. Actuators B Chem..

[306] Chen Q., Li H., Ouyang Q., Zhao J. (2014). Identification of spoilage bacteria using a simple colorimetric sensor array. Sens. Actuators B Chem..

[307] Sheini A. (2021). A point-of-care testing sensor based on fluorescent nanoclusters for rapid detection of septicemia in children. Sens. Actuators B Chem..

[308] Ali M.M., Brown C.L., Jahanshahi-Anbuhi S., Kannan B., Li Y., Filipe C.D.M., Brennan J.D. (2017). A Printed Multicomponent Paper Sensor for Bacterial Detection. Sci. Rep..

[309] Sun Y., Chang Y., Zhang Q., Liu M. (2019). An origami paper-based device printed with DNAzyme-containing DNA superstructures for Escherichia Coli detection. Micromachines.

[310] Sun Q., Tam N.F.Y., Han J., Yung-Kang Peng W., Zhu Z., Chen J.L. (2021). A simple paper-based colorimetric analytical device for rapid detection of Enterococcus faecalis under the stress of chlorophenols. Talanta.

[311] Silva N.F.D., Almeida C.M.R., Magalhães J.M.C.S., Gonçalves M.P., Freire C., Delerue-Matos C. (2019). Development of a disposable paper-based potentiometric immunosensor for real-time detection of a foodborne pathogen. Biosens. Bioelectron..

[312] Rengaraj S., Cruz-Izquierdo Á., Scott J.L., Di Lorenzo M. (2018). Impedimetric paper-based biosensor for the detection of bacterial contamination in water. Sens. Actuators B Chem..

[313] Unnikrishnan B., Lien C.-W., Chu H.-W., Huang C.-C. (2021). A review on metal nanozyme-based sensing of heavy metal ions: Challenges and future perspectives. J. Hazard. Mater..

[314] Malik L.A., Bashir A., Qureashi A., Pandith A.H. (2019). Detection and removal of heavy metal ions: A review. Environ. Chem. Lett..

[315] Sall M.L., Diaw A.K.D., Gningue-Sall D., Efremova Aaron S., Aaron J.J. (2020). Toxic heavy metals: Impact on the environment and human health, and treatment with conducting organic polymers, a review. Environ. Sci. Pollut. Res..

[316] Ninwong B., Sangkaew P., Hapa P., Ratnarathorn N., Menger R.F., Henry C.S., Dungchai W. (2020). Sensitive distance-based paper-based quantification of mercury ions using carbon nanodots and heating-based preconcentration. RSC Adv..

[317] Chen G.H., Chen W.Y., Yen Y.C., Wang C.W., Chang H.T., Chen C.F. (2014). Detection of mercury(II) ions using colorimetric gold nanoparticles on paper-based analytical devices. Anal. Chem..

[318] Chen W., Fang X., Li H., Cao H., Kong J. (2016). A Simple Paper-Based Colorimetric Device for Rapid Mercury(II) Assay. Sci. Rep..

[319] Cai L., Fang Y., Mo Y., Huang Y., Xu C., Zhang Z., Wang M. (2017). Visual quantification of Hg on a microfluidic paper-based analytical device using distance-based detection technique. AIP Adv..

[320] Nashukha H.L., Sitanurak J., Sulistyarti H., Nacapricha D., Uraisin K. (2021). Simple and equipment-free paper-based device for determination of mercury in contaminated soil. Molecules.

[321] Quinn C.W., Cate D.M., Miller-Lionberg D.D., Reilly T., Volckens J., Henry C.S. (2018). Solid-Phase Extraction Coupled to a Paper-Based Technique for Trace Copper Detection in Drinking Water. Environ. Sci. Technol..

[322] Ratnarathorn N., Chailapakul O., Henry C.S., Dungchai W. (2012). Simple silver nanoparticle colorimetric sensing for copper by paper-based devices. Talanta.

[323] Wang X., Sun J., Tong J., Guan X., Bian C., Xia S. (2018). Paper-based sensor chip for heavy metal ion detection by SWSV. Micromachines.

[324] Fang X., Zhao Q., Cao H., Liu J., Guan M., Kong J. (2015). Rapid detection of Cu^2+^ by a paper-based microfluidic device coated with bovine serum albumin (BSA)-Au nanoclusters. Analyst.

[325] Wang H., Da L., Yang L., Chu S., Yang F., Yu S., Jiang C. (2020). Colorimetric fluorescent paper strip with smartphone platform for quantitative detection of cadmium ions in real samples. J. Hazard. Mater..

[326] WHO (2008). Guidelines for Drinking-water Quality.

[327] López Marzo A.M., Pons J., Blake D.A., Merkoçi A. (2013). All-integrated and highly sensitive paper based device with sample treatment platform for Cd^2+^ immunodetection in drinking/tap waters. Anal. Chem..

[328] Huang K., Chen Y., Zhou F., Zhao X., Liu J., Mei S., Zhou Y., Jing T. (2017). Integrated ion imprinted polymers-paper composites for selective and sensitive detection of Cd(II) ions. J. Hazard. Mater..

[329] EPA 816-F-09-0004, United States Environmental Protection Agency https://www.nrc.gov/docs/ML1307/ML13078A040.pdf.

[330] Gupta A., Verma N.C., Khan S., Tiwari S., Chaudhary A., Nandi C.K. (2016). Paper strip based and live cell ultrasensitive lead sensor using carbon dots synthesized from biological media. Sens. Actuators B Chem..

[331] Wang H., Yang L., Chu S., Liu B., Zhang Q., Zou L., Yu S., Jiang C. (2019). Semiquantitative Visual Detection of Lead Ions with a Smartphone via a Colorimetric Paper-Based Analytical Device. Anal. Chem..

[332] Pechova A., Pavlata L. (2007). Chromium as an essential nutrient: A review. Vet. Med..

[333] Elavarasi M., Rajeshwari A., Chandrasekaran N., Mukherjee A. (2013). Simple colorimetric detection of Cr(III) in aqueous solutions by as synthesized citrate capped gold nanoparticles and development of a paper based assay. Anal. Methods.

[334] Feng L., Li H., Niu L.Y., Guan Y.S., Duan C.F., Guan Y.F., Tung C.H., Yang Q.Z. (2013). A fluorometric paper-based sensor array for the discrimination of heavy-metal ions. Talanta.

[335] Feng L., Li X., Li H., Yang W., Chen L., Guan Y. (2013). Enhancement of sensitivity of paper-based sensor array for the identification of heavy-metal ions. Anal. Chim. Acta.

[336] Devadhasan J.P., Kim J. (2018). A chemically functionalized paper-based microfluidic platform for multiplex heavy metal detection. Sens. Actuators B Chem..

[337] Zhang L., Guan L., Lu Z., Li M., Wu J., Cao R., Tian J. (2019). Barrier-free patterned paper sensors for multiplexed heavy metal detection. Talanta.

[338] Wu J., Li M., Tang H., Su J., He M., Chen G., Guan L., Tian J. (2019). Portable paper sensors for the detection of heavy metals based on light transmission-improved quantification of colorimetric assays. Analyst.

[339] Muhammad-aree S., Teepoo S. (2020). On-site detection of heavy metals in wastewater using a single paper strip integrated with a smartphone. Anal. Bioanal. Chem..

[340] Qi J., Li B., Wang X., Zhang Z., Wang Z., Han J., Chen L. (2017). Three-dimensional paper-based microfluidic chip device for multiplexed fluorescence detection of Cu^2+^ and Hg^2+^ ions based on ion imprinting technology. Sens. Actuators B Chem..

[341] Hossain S.M.Z., Brennan J.D. (2011). β-Galactosidase-based colorimetric paper sensor for determination of heavy metals. Anal. Chem..

[342] Wang X.R., Li B.W., You H.Y., Chen L.X. (2015). An ion imprinted polymers grafted paper-based fluorescent sensor based on quantum dots for detection of Cu^2+^ ions. Chin. J. Anal. Chem..

[343] He K., Zhan X., Liu L., Ruan X., Wu Y. (2020). Ratiometric Fluorescent Paper-Based Sensor Based on CdTe Quantum Dots and Graphite Carbon Nitride Hybrid for Visual and Rapid Determination of Cu^2+^ in Drinks. Photochem. Photobiol..

[344] Zhang M., Ge L., Ge S., Yan M., Yu J., Huang J., Liu S. (2013). Three-dimensional paper-based electrochemiluminescence device for simultaneous detection of Pb^2+^ and Hg^2+^ based on potential-control technique. Biosens. Bioelectron..

[345] Shi J., Tang F., Xing H., Zheng H., Bi L., Wang W. (2012). Electrochemical detection of Pb and Cd in paper-based microfluidic devices. J. Braz. Chem. Soc..

